# Metabololipidomic and proteomic profiling reveals aberrant macrophage activation and interrelated immunomodulatory mediator release during aging

**DOI:** 10.1111/acel.13856

**Published:** 2023-04-26

**Authors:** Patrick Schädel, Anna Czapka, Nadja Gebert, Ilse Denise Jacobsen, Alessandro Ori, Oliver Werz

**Affiliations:** ^1^ Department of Pharmaceutical/Medicinal Chemistry, Institute of Pharmacy Friedrich‐Schiller University Jena Germany; ^2^ Leibniz Institute for Natural Product Research and Infection Biology – Hans Knoell Institute (HKI) Jena Germany; ^3^ Leibniz Institute on Aging – Fritz Lipmann Institute (FLI) Jena Germany; ^4^ Institute of Microbiology Friedrich‐Schiller University Jena Germany

**Keywords:** aging, eicosanoids, inflammation, lipidomics, macrophage activation, mediators of inflammation, peritoneal macrophages, proteomics

## Abstract

Macrophages adapt distinct pro‐inflammatory (M1‐like) and pro‐resolving (M2‐like) phenotypes with specific tasks in the immune response and tissue homeostasis. Altered macrophage responses with age are causative for unresolved inflammation, so‐called inflammaging, and lead to higher infection susceptibility with unfavorable progression. Here, we reveal molecular determinants of age‐related changes in phenotypic functions of murine peritoneal macrophages (PM) by employing comprehensive mass spectrometry‐based proteomics (4746 protein groups) and metabololipidomics (>40 lipid mediators). Divergent expression of various macrophage‐specific marker proteins and signaling pathways indicates aberrant PM phenotypes in old mice which detrimentally impact their capabilities to release immunomodulatory chemokines and cytokines. We show that aging strikingly compromises the polarization process of macrophages to adapt either pro‐inflammatory or pro‐resolving phenotypes, thereby yielding aberrant and afunctional macrophage subtypes that cannot be readily assigned to either a typical M1 or M2 phenotype. In particular, the phenotypic adaptation of the bacteria‐challenged metabololipidome in macrophages related to inflammation is severely limited by age, which persists across ex vivo polarization towards M1 and M2a macrophages. Our results establish distinct age‐associated PM phenotypes outside of the simplified M1 and M2 dichotomy and challenge the dogma of increased pro‐inflammatory macrophage pre‐activation due to aging by revealing maladaptive functions throughout all phases of inflammation, including resolution.

## Graphical abstract

Aging impacts macrophage polarization towards pro‐inflammatory (M1) or pro‐resolving phenotypes (M2a). We show that the metabololipidome of peritoneal macrophages prior and after ex vivo polarization is limited by age, affecting initiation and resolution of inflammation. Proteome profiling revealed distinct age‐associated phenotypes outside of the simplified M1 and M2 dichotomy, strengthening the need to expand classification to address age‐related aberrations in their immunological competence.

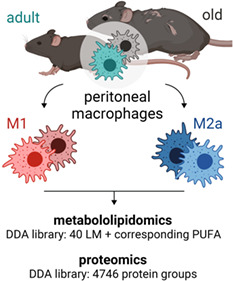

**Abbreviations**
AA: arachidonic acid
BCA: Bcell‐attracting chemokine
BMDM: bone marrow‐derived macrophage
CD: clusterof differentiation
COX: cyclooxygenase
cPGES: cytosolic prostaglandin Esynthase
cPLA_2_
: cytosolic phospholipase A_2_
CXCL: C‐X‐C motif chemokine
DDA: data‐dependent analysis
DHA: docosahexaenoicacid
DIA: data‐independent analysis
EPA: eicosapentaenoic acid
FLAP: 5‐lipoxgenase‐activating protein
HDHA: hydroxy‐docosahexaenoic acid
HEPE: hydroxy‐pentaenoic acid
HETE: hydroxy‐tetraenoic acid
HODE: hydroxy‐octadecadienoic acid
IFN: interferon
IL: interleukin
IP‐10: interferon‐gammainduced protein 10 kD
KC: Platelet‐derivedgrowth factor‐inducible protein KC
LM: lipid mediator
LOX: lipoxygenase
LPS: lipopolysaccharide
LT: leukotriene
LTA_4_H: leukotriene A_4_ hydrolase
LX: lipoxin
MaR: maresin
MCP: monocyte chemoattractant protein
MDC: macrophage‐derived chemokine
MFI: mean fluorescence intensity
MOI: multiplicity of infection
mPGES: microsomal prostaglandin E synthase
NO: nitric oxide
PAMP: pathogen‐associated molecular pattern
PCA: principalcomponent analysis
PDX: protectin
PG: prostaglandin
PGFS: prostaglandin Fsynthase
PM: peritoneal macrophage
PTGIS: prostaglandin I synthase
PUFA: polyunsaturatedfatty acid
ROS: reactive oxygen species
RvD: D‐series resolvin
RvE: E‐series resolvin
SM: splenic macrophage
SPM: specialized pro‐resolvingmediator
TBXAS: thromboxane A synthase
TGF: tumor growth factor
TNF: tumor necrosis factor
UPLC‐MS/MS: ultra‐high performance liquidchromatography‐coupled tandem mass spectrometry

## 1 INTRODUCTION

Although the life expectancy for humans markedly increased in the last decades and is projected to rise further, this positive trend is, however, accompanied by a higher susceptibility to inflammatory and infectious diseases in elderly individuals (Franceschi et al., 2018). Immunosenescence is widely regarded as the root cause for the age‐associated impairment in the efficiency of the immune response (Aiello et al., 2019). One major hallmark is inflammaging, which is classified as a systemic, chronic, and low‐grade inflammation impairing tissue homeostasis and repair during aging (Franceschi et al., 2018). In this context, macrophages play a major role as regulatory innate immune cells which, by releasing immunomodulatory mediators, are involved in initiation, perpetuation, and resolution of inflammatory processes (Okabe & Medzhitov, 2016). Macrophages engulf and digest invading pathogens, clear up debris from injury and/or orchestrate the inflammatory response through chemical attraction of other effector immune cells like monocytes and neutrophils (Arango Duque & Descoteaux, 2014; Price & Vance, 2014). Furthermore, they are an important linkage to the adaptive immune system through the presentation of antigens, which are recognized by T cells (Gaudino & Kumar, 2019).

During inflammation, macrophages constantly adapt their inflammatory (M1) and pro‐resolving (M2) phenotypes to the surrounding microenvironment and adjust the release of immunomodulatory mediators such as cytokines, chemokines, and lipid mediators (LM) (Lavin et al., 2015; Murray, 2020). The release of different types of LM from activated macrophage populations regulates all phases of inflammation, from the onset to the resolution (Serhan, 2014). M1 macrophages efficiently eliminate pathogens (Ley, 2017) and mainly generate pro‐inflammatory prostaglandins (PG) and leukotrienes (LT) from polyunsaturated fatty acids (PUFA) liberated by phospholipases A_2_ via cyclooxygenase (COX) and 5‐lipoxygenase (5‐LOX) pathways, respectively (Dalli & Serhan, 2012; Werz et al., 2018). Thereby, they cause increased blood‐flow and recruitment of neutrophils as initial countermeasures to infection or damage (Funk, 2001). Upon pathogen clearance, macrophages actively promote tissue repair and return to homeostasis through adaptation of a pro‐resolving (M2) phenotype (Okabe & Medzhitov, 2014; Watanabe et al., 2019). This macrophage phenotype switch is accompanied by a shift in the formation of pro‐inflammatory PG and LT towards the superfamily of specialized pro‐resolving mediators (SPM) that encompass lipoxins, resolvins, maresins, and protectins which are crucial to drive resolution of inflammation (Dalli & Serhan, 2012; Serhan & Savill, 2005; Werz et al., 2018).

Due to their extensive plasticity, macrophages are prone to age‐associated changes within the inflammatory microenvironment, termed macrophaging (Prattichizzo et al., 2016). Several aging‐driven defects in macrophage polarization were reported, but conclusive age‐related phenotypes remain elusive. Thus, observations include decreased polarization markers in peritoneal macrophages (PM) (Linehan et al., 2014), altered phagocytic activity of Kupffer cells and alveolar macrophages (Hilmer et al., 2007; Linehan et al., 2014; Wong et al., 2017), and a generally increased pro‐inflammatory phenotype of bone‐marrow derived macrophages (BMDM) and Kupffer cells of old origin (Becker et al., 2018; Gibon et al., 2016). However, in‐depth characterization of the phenotypic changes of macrophages with age and evaluation of their capabilities to adapt to the dynamic inflammatory microenvironment are missing.

Here, we provide substantial insights into age‐associated aberrations of the proteome and metabololipidome of murine tissue‐resident PM and their distinct phenotypes with concomitant alterations in the release of immunomodulatory mediators. We employed comprehensive metabololipidomic and proteomic profiling of PM from adult and old mice to reveal age‐related changes of phenotypic markers, signaling and activation pathways, and release of cytokines/chemokines and LM. Our results indicate a deprivation of inflammatory pathways in old PM, which persists across ex vivo polarization and impairs the temporal release of immunomodulatory mediators during bacterial infection and the clearance of bacterial debris.

## 2 RESULTS

### 2.1 Aging increases resident PM numbers and impacts macrophage activation markers

**FIGURE 1:**
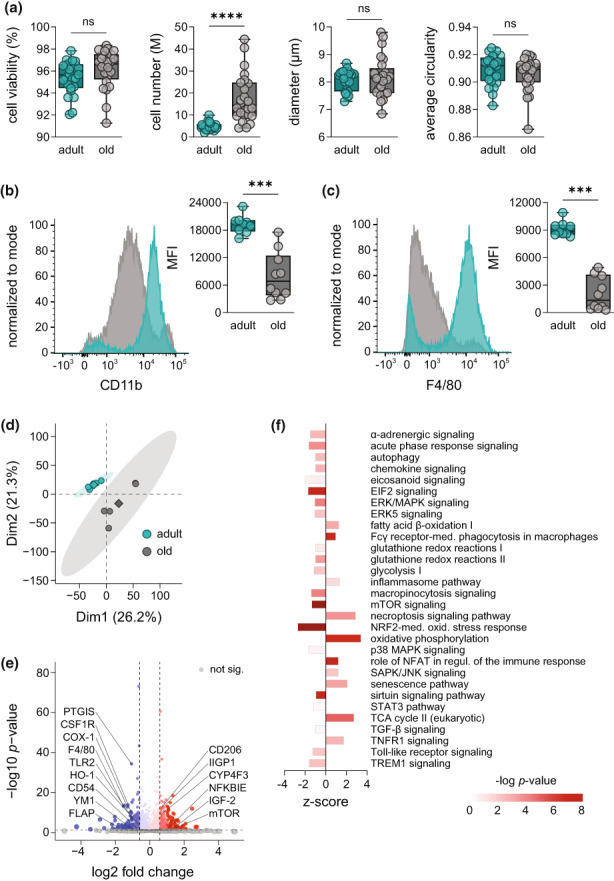
Aging establishes a distinct macrophage phenotype in the peritoneal cavity. (a) Number, viability, diameter, and circularity of isolated cells from the peritoneal cavity of adult (4–6 months) and old mice (>24 months) were assessed with a Vi‐CELL XR system (*n* = 25). Expression of surface markers (b) CD11b and (c) F4/80 on PM from adult (turquoise) and old mice (grey) was measured by flow cytometry (*n* = 9–10). Results are shown in representative histograms for both surface markers with mean fluorescence intensity (MFI) of all replicates. (d) Principal component analysis (PCA) of the proteome of PM from adult and old mice measured by DIA mass spectrometry (*n* = 5). (e) Volcano plot displays proteins significantly increased or decreased by aging (Table S1; *n* = 5). Not affected proteins are shown in grey. Dashed lines indicate a cutoff for significance of *p* < 0.05 and absolute fold changes (log_2_) > 0.58, respectively. Significantly regulated proteins of interest were labelled with an emphasis on those involved in macrophage function during inflammation. (f) Ingenuity pathway analysis of significantly regulated protein clusters in PM from old mice in comparison with adult (*n* = 5). Displayed pathways are among the Top 100 most significantly regulated pathways and were selected based on the relevance for aging and inflammation. Bonferroni–Holm corrected *p*‐values are implicated by color and *z*‐scores by bar size. Statistics: Data are shown as (a–c): median (min to max) or (e, f) median and *p*‐values were calculated by ‘a–c’ unpaired two‐tailed Student's *t*‐test with or without Welch's correction (Table S7), (e) Spectronaut™ (Table S1) or (f) QIAGEN Ingenuity Pathway Analysis. ***p ≤ 0.001, ****p ≤ 0.0001, ns, not significant.

To study how aging affects the composition and phenotype of macrophages within the murine peritoneal cavity, we isolated resident peritoneal cells from adult (4–6 months) and old (>24 months) C57BL/6JRj mice by peritoneal lavage. Cells from both age cohorts were similar in terms of viability, diameter, and average circularity (Figure 1a). The cell number significantly increased from averagely 4.48 to 13.15 × 10^6^ cells as consequence of aging (Figure 1a). To confirm that after initial seeding PM are the most abundant cell type, we assessed CD11b and F4/80 as surface markers (Ghosn et al., 2010) by flow cytometry. Regardless of the age, most cells (83.2% to 89.1%) were positive for both markers, with slightly higher portion of CD11b^+^/F4/80^−^ cells from old mice (Figure S1a). Notably, the mean fluorescence intensity (MFI) for both CD11b and F4/80 was strongly reduced as consequence of aging (Figure 1b,c), confirming different origin of macrophages (Bain et al., 2016). To study if aging affects the functionality of PM, we assessed their phagocytic capacity for engulfing fluorescent‐labelled *E. coli* particles and found no difference between both age cohorts (Figure S1b). Interestingly, we found that treatment of PM from adult mice for 6 h with the supernatant of LPS‐challenged (18 h) old (but not adult) PM led to a significant reduction in phagocytosis (Figure S1c). This indicates that the secretome of adult and old PM has divergent impact on macrophage functions.

We then investigated how aging affects activation markers and interrelated inflammatory signaling pathways in PM by employing mass spectrometry (MS)‐based proteomic and metabololipidomic profiling. Comprehensive libraries for both the proteome (DDA library: 4746 protein groups) and the metabololipidome (DDA library: 40 LM and corresponding PUFA) of PM from both age cohorts were identified (Figure S1d). Principal component analysis (PCA) of the overall proteome revealed striking differences between age groups as indicated by clearly defined, separated clusters for adult and old PM (Figure 1d). Among the most strikingly regulated proteins by aging, we found several macrophage activation markers and key regulatory enzymes in inflammation (Figure 1e). Thus, reduced amounts of intercellular adhesion molecule 1 (CD54) that mediates homing and trafficking of inflammatory cells to distant tissues, or chitinase‐like protein 3 (YM1) with chemotactic activity for eosinophils, in old PM (Figure 1e) implicate decreased activation states. Interestingly, the levels of the LM‐biosynthetic proteins COX‐1, prostacyclin synthase (PTGIS), and 5‐LOX‐activating protein (FLAP) were strongly reduced (>twofold) in old PM (Figure 1e), suggesting a close association between aging and the formation of inflammatory LM produced by these enzymes. In contrast, various signaling proteins, for example, mTOR, IGF‐2, and NFKBIE were elevated due to aging (Figure 1e). The proteome data also confirmed the reduced levels of CD11b and F4/80 in old PM (assessed by flow cytometry, see above) alongside decreased CD14 expression (Figure S1e).

To provide a concise overview of signaling pathways affected in PM by aging, we exploited ingenuity pathway analysis (IPA; Qiagen) to cluster the proteomic data. Among the top‐100 affected pathways were several of those that are pivotal for macrophage activation and inflammatory signaling (Figure 1f). Proteins connected to signaling of eicosanoids, EIF2, ERK/MAPK, mTOR, Toll‐like receptor, TREM1 and micropinocytosis as well as the nuclear factor erythroid 2‐related factor 2 (NRF‐2)‐mediated oxidative response were markedly downregulated in old PM (Figure 1f), while proteins related to the role of Fcγ receptor (phagocytosis), NFAT (immune response), necroptosis signaling, and oxidative phosphorylation were elevated (Figure 1f). Furthermore, senescence signaling was upregulated while the sirtuin cascade was impaired, both of which are associated with aging (Imperatore et al., 2017; Prattichizzo et al., 2016). Together, these results imply a distinct macrophage phenotype for old PM, potentially connected to altered immune responses against pathogens and inflammatory signaling.

### 2.2 Downregulation of COX‐1 and FLAP during aging compromises PG and LT formation

**FIGURE 2:**
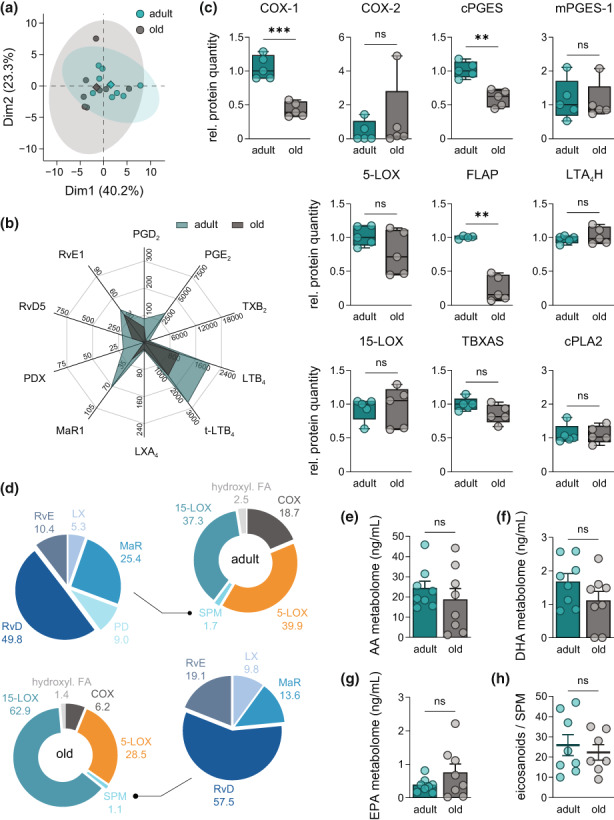
Aging compromises the metabololipidome of *E. coli*‐infected peritoneal macrophages. (a) PCA of the metabololipidomic profile of PM from adult and old mice after *E. coli* infection (Table S6; *n* = 8). (b) Radar chart of selected bioactive LM released by PM of adult and old origin after *E. coli* infection (Table S6; *n* = 8; unit: pg/mL). (c) Relative abundance of COX‐1, COX‐2, cPGES, mPGES‐1, 5‐LOX, FLAP, LTA_4_H, 15‐LOX, TBXAS, and cPLA_2_ in PM from adult and old mice, measured by DIA mass spectrometry (*n* = 5). (d) Composition of the metabololipidome of adult and old PM after *E. coli* infection (Table S6; *n* = 8). Metabolites were grouped as follows: *COX‐2* – PGD_2_, PGE_2_, PGF_2α_, TXB_2_; PGE_1_; *5‐LOX* – LTB_4_, *t*‐LTB_4_, epi *t*‐LTB_4_, 5‐HETE, 5‐HEPE, 5S,6R‐diHETE; *SPM* – LXA_4_, AT‐LXA_4_, LXA_5_, LXB_4_, MaR1, MaR2, PD1, PDX, RvD1, AT‐RvD1, RvD2, RvD3, RvD4, RvD5, RvE1; *15‐LOX* – 14‐HDHA, 17‐HDHA, 12‐HEPE, 15‐HEPE, 12‐HETE, 15‐HETE, 5,15‐diHETE; *hydroxylated FA* – 4‐HDHA, 7‐HDHA, 10‐HDHA, 13‐HDHA, 11‐HEPE, 18‐HEPE, 8‐HETE, 11‐HETE, 9‐HODE, 13‐HODE. Fatty acid metabolomes are given as sum of all metabolites from (e) AA, (f) DHA and (g) EPA released by PM of adult and old mice after infection with pathogenic *E. coli* (Table S6; *n* = 8). (h) Ratio of pro‐inflammatory PGE_2_, LTB_4_, *t*‐LTB_4,_ and epi *t*‐LTB_4_ to all SPM (see above) released by adult and old PM after *E. coli* infection (Table S6; *n* = 8). Statistics: Data are shown as (b, d) mean or (c) median (min to max) or (e–h) mean ± SEM and *p*‐values were calculated by unpaired two‐tailed Student's *t*‐test with or without Welch's correction (Table S7). **p ≤ 0.01, ***p ≤ 0.001, ns, not significant.

Production of either LT and PG or SPM upon macrophage challenge with bacteria distinguishes their pro‐inflammatory or pro‐resolving characteristics, respectively (Werz et al., 2018). To study how aging impacts the response of PM to bacterial challenge, we infected adult and old PM with pathogenic *E. coli* (MOI of 50) and performed comprehensive metabololipidomic profiling. PCA indicated similar LM signatures for both PM cohorts with only minor deviations (Figure 2a). Yet, closer inspection of single bioactive LM revealed marked differences as consequence of aging. Most strikingly, PGE_2_ formation was strongly reduced from 3325 to 247 pg/mL, but also the levels of pro‐inflammatory LTB_4_ (2024–1083 pg/mL) and *trans*‐LTB_4_ (3278–1819 pg/mL) were impaired, in old PM (Figure 2b). Interestingly, besides these pro‐inflammatory LM also SPM like maresin‐1 (MaR1) from 72.7 to 27.3 pg/mL and protectin DX (PDX) from 20.2 pg/mL to non‐detectable (<3 pg/mL) amounts were lowered (Figure 2b).

Assessment of LM‐biosynthetic enzymes in the PM proteome showed that levels of FLAP and COX‐1 were lower in old PM, correlating to impaired LTB_4_ and PGE_2_ formation, respectively (Figure 2c). In line with diminished COX‐1 expression, the amounts of the functionally coupled cytosolic PGE synthase (cPGES) dropped significantly (Figure 2c). In contrast, the protein levels of 5‐ and 15‐LOX, LTA_4_ hydrolase (LTA_4_H), COX‐2, microsomal PGE synthases (mPGES)‐1/2, PGF synthase (PGFS), thromboxane A synthase (TXAS), or cytosolic PLA_2_ (cPLA_2_) were not changed with aging (Figure 2c).

Next, we analyzed whether aging impacts the overall composition of bacteria‐induced LM signature profiles by clustering all detectable LM according to their biosynthetic pathways. Aging impaired formation of COX‐ and 5‐LOX‐derived products from 18% to 6% and 40.3% to 28.5% of total LM, respectively (Figure 2d). The aging‐induced drop of SPM from 1.7 to 1.0% is mainly due to markedly reduced protectin and maresin formation (Figure 2c,d). In contrast, the fraction of 15‐LOX‐derived SPM precursors increased from 37.6% to 63.1% due to aging (Figure 2d). The changes in the metabololipidome were not based on an altered availability of PUFAs since levels of arachidonic acid (AA)‐, eicosapentaenoic acid (EPA)‐ and docosahexaenoic acid (DHA)‐derived LM were not affected by age (Figure 2e–g). Finally, the ratio of pro‐inflammatory eicosanoids to pro‐resolving SPM was not significantly affected by age (Figure 2h). In conclusion, we revealed age‐related changes of the metabololipidome of *E. coli*‐infected PM, with impaired responses of old PM to pathogenic bacteria.

### 2.3 Aging alters the macrophage phenotype of naive tissue‐resident macrophages

**FIGURE 3:**
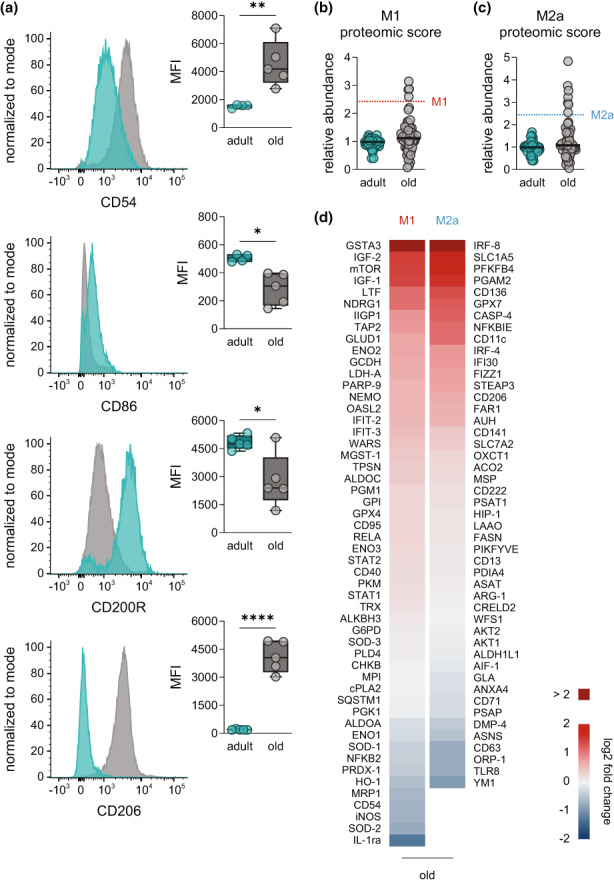
Investigation of polarization markers in naive peritoneal macrophages. (a) Expression of M1 (CD54, CD86) and M2a surface markers (CD200R, CD206) in naive PM from adult and old mice was determined by flow cytometry. Results are shown as representative histograms with MFI of all replicates (*n* = 4–5). Abundance of (b) M1 and (c) M2a proteomic markers in PM from adult and old mice was determined by DIA mass spectrometry. Each dot represents the median of a different marker (the complete list is given in Table S2) and median of all markers is indicated by vertical bars, whereas dotted lines show median in M1‐ and M2a‐PM (48 h) from adult mice (*n* = 5). (d) Heatmap showing age‐related changes in the expression of M1 and M2a proteomic markers in PM from old mice in comparison with adult (*n* = 5). Fold change is implicated by color scale. Statistics: Data are shown as (a) median (min to max) or (b–d) as median and *p*‐values were calculated by unpaired two‐tailed Student's *t*‐test with or without Welch's correction (Table S7). *p ≤ 0.05, **p ≤ 0.01, ****p ≤ 0.0001.

To study how aging affects the polarization state of naive PM resident in the peritoneal cavity, we first assessed surface markers that are generally used to define pro‐inflammatory M1 (CD54, CD86) or pro‐resolving M2a (CD200R, CD206) phenotypes in mice using flow cytometry (Schulz et al., 2019). PM from old mice displayed higher levels of CD54 (ICAM‐1), while CD86 (B7‐2) was overall weakly expressed in PM with lower levels in cells from old mice (Figure 3a). Interestingly, CD200R was reduced due to aging, while CD206 was strikingly increased (Figure 3a). These findings essentially agree with the proteomic analysis, although differences of CD54 in old versus adult PM did not correlate (Figure S1f,g). Additionally, aging altered the levels of intracellular marker proteins associated with M1 (iNOS) and M2a polarization (ARG‐1, FIZZ1, YM1), even though not significantly (Figure S1h,i). For in‐depth investigation of the PM polarization state, we selected over 50 protein markers that are characteristic for either M1‐like or M2a‐like macrophages (Table S2), which align with established gene signatures of the respective macrophage phenotypes (Jablonski et al., 2015; Orecchioni et al., 2019). We found that PM from both age groups did not exhibit a marker profile corresponding to either classical M1‐ or M2a‐polarization (Figure 3b,c). Enrichment analysis further underlined this finding showing no significant upregulation towards the M1 marker set (score: 1.3578; *p* = 0.0863) and just a minor increase in M2a markers (score: 1.6014; *p* = 0.0109), which hardly resembles physiological polarization towards M2a. By comparing the levels of individual proteins from both marker sets between old and adults PM, most of them were differentially regulated (Figure 3d). Most strikingly, M1 markers such as IL‐1ra, SOD‐2, iNOS, and CD54, and M2a markers like YM1, TLR8, ORP‐1, and CD63 were downregulated in PM of old mice. In contrast, GSTA3, IGF‐1, IGF‐2, and mTOR (M1 markers) and IRF‐8, SLC1A5, PFKFB4, and PGAM2 (M2a markers) were upregulated due to aging.

To examine whether observed age‐related changes in polarization and the metabololipidome of PM apply also to macrophages from other immunologic niches, we performed side‐by‐side analysis of PM with splenic macrophages (SM) and BMDM from the same mice. PCA of the metabololipidome after *E. coli* infection revealed distinct phenotypes for both SM and BMDM as consequence of aging (Figure S3a). Interestingly, we found an age‐driven downregulation of COX‐derived PG and TX in all macrophage species (Figure S3b–d) in line with results shown in Figure 2b. Yet, the metabolipidome strongly varied between PM, SM, and BMDM both in the overall amounts of produced LM and in the composition of the LM species (Table S6). SM mainly released PG, TXB_2_, LT and moderate levels of RvD5 and RvE1 (Figure S3d), while BMDM produced low amounts of LM, mainly TXB_2_ (Figure S3c). Furthermore, we found significant downregulation of F4/80 and CD54 and a downward trend for CD86 and CD200R in all three macrophage types from different niches (Figure S3e). Together, our data imply distinct macrophage polarization states of adult and old macrophages, with neither a classical M1‐ nor M2a‐like phenotype, but clearly differing between the age cohorts.

### 2.4 Impairment of functional polarization of PM as consequence of aging

**FIGURE 4:**
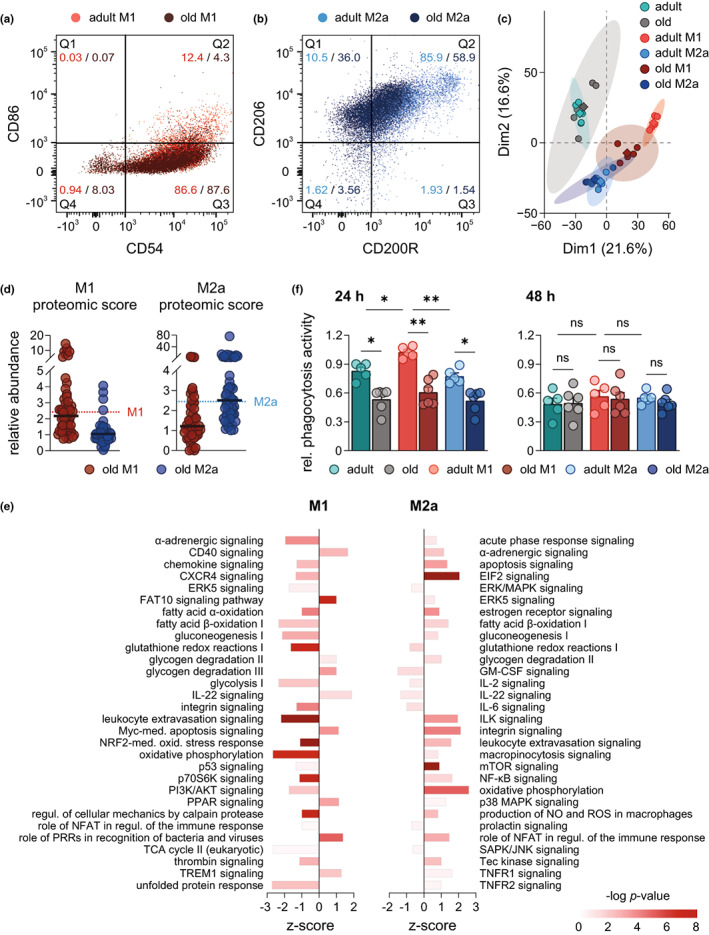
Influence of aging on the phenotype and functionality of polarized peritoneal macrophages. Expression of (a) M1 (CD54, CD86) and (b) M2a (CD200R, CD206) surface markers in polarized PM (48 h) was determined by flow cytometry, and results are shown in a representative overlaid dot plot indicating percental distribution of populations (*n* = 5). (c) PCA of the proteome of PM from adult and old mice after 48 h of polarization measured by DIA mass spectrometry (*n* = 5). (d) Abundance of M1 and M2a proteomic markers in polarized PM (48 h) of old mice was determined by DIA mass spectrometry. Each dot represents the median of a different marker (complete list is given in Table S2) and median of all markers is indicated by vertical bars, whereas dotted lines show median in M1‐ and M2a‐PM (48 h) from adult mice (*n* = 5). (e) Ingenuity pathway analysis of significantly regulated protein clusters in M1 and M2a‐PM (48 h) from old mice in comparison with adult (*n* = 5). Displayed pathways are among the Top 100 most significantly regulated pathways and were selected based on their relevance for aging and inflammation. Bonferroni–Holm corrected *p*‐value is implicated by color and z‐score by bar size. (f) Naive and polarized PM (24 h and 48 h) from adult and old mice were incubated with fluorescent‐labelled *E. coli* particles for 2 h, and phagocytic activity was determined by measuring fluorescence after uptake (*n* = 5–6). Statistics: Data are shown as (d, e) median or (f) mean ± SEM and *p*‐values were calculated by (e) QIAGEN Ingenuity Pathway Analysis or (f) one‐way ANOVA for multiple comparisons with Šídák post‐hoc test or Brown‐Forsythe and Welch ANOVA with Dunnett T3 post‐hoc test (Table S7).

To elucidate whether aging affects the polarization process of PM, we polarized naive PM from adult and old mice ex vivo for 48 h using lipopolysaccharide (LPS) plus interferon (IFN)‐γ (M1‐PM) and interleukin (IL)‐4 (M2a‐PM), respectively (Murray et al., 2014). Analysis by flow cytometry revealed no significant age‐related changes in the levels of M1 markers CD54 and CD86 in M1‐PM (Figure 4a, Figure S4a). However, expression of M2a markers differed significantly in adult and old M2a‐PM, where 85.9% of adult cells were positive for both CD200R and CD206, which dropped to 58.9% in old M2a‐PM (Figure 4b). Specifically, CD200R levels were significantly lower in old M2a‐PM, while CD206 was not affected (Figure S4a). Proteomic profiling of the PM and PCA of the overall proteome showed that polarized adult and old PM developed distinct M1‐ and M2a‐like phenotypes, with aging impacting the clear separation of both polarization states (Figure 4c). Quantification of a wide range of M1 and M2a phenotypic markers revealed a slight drop in the median of M1 markers in old M1‐PM, while the median expression of M2a markers was not affected (Figure 4d). Comparison of each protein marker individually between both age cohorts revealed distinct phenotypes as consequence of aging: M1‐PM mostly downregulated typical M1 markers in favor of elevated M2a markers, while in M2a‐PM both marker sets showed elevation and reduction (Figure S4b). IPA of the overall proteome confirmed aging‐induced divergent activation of multiple inflammation‐associated pathways in M1‐ and M2a‐PM from both age cohorts (Figure 4e). Overall, aging downregulated most pathways in M1‐PM including glycolysis, NRF‐2, and hypoxia‐inducible factor 1‐alpha (HIF‐1α) signaling (Figure 4e), which are necessary for the inflammatory function of M1 macrophages (Kobayashi et al., 2016; Viola et al., 2019). In contrast, pro‐inflammatory pathways involving acute phase response signaling, integrin signaling, mTOR, NF‐κB, NO and ROS production, p38 MAPK and TNFR1/2 signaling were significantly upregulated in old M2a‐PM (Figure 4e). Together, aging markedly impacts ex vivo polarization of PM yielding aberrant M1 and M2a phenotypes, with apparent shifts in the activation of inflammation‐associated signaling pathways. To elucidate whether the age‐associated changes in the polarized PM phenotypes affect their function, we assessed their capacities for phagocytosis. Interestingly, after 24 h the phagocytic activity of old PM was significant reduced regardless of the polarization state, while no changes were apparent at the later timepoint of 48 h (Figure 4f). Of note, the phagocytic capacity of adult M1‐PM was significantly higher over unpolarized or M2a‐PM of the same age cohort (Figure 4f).

### 2.5 Aging impacts the capacities of polarized PM for chemokine and cytokine release

**FIGURE 5:**
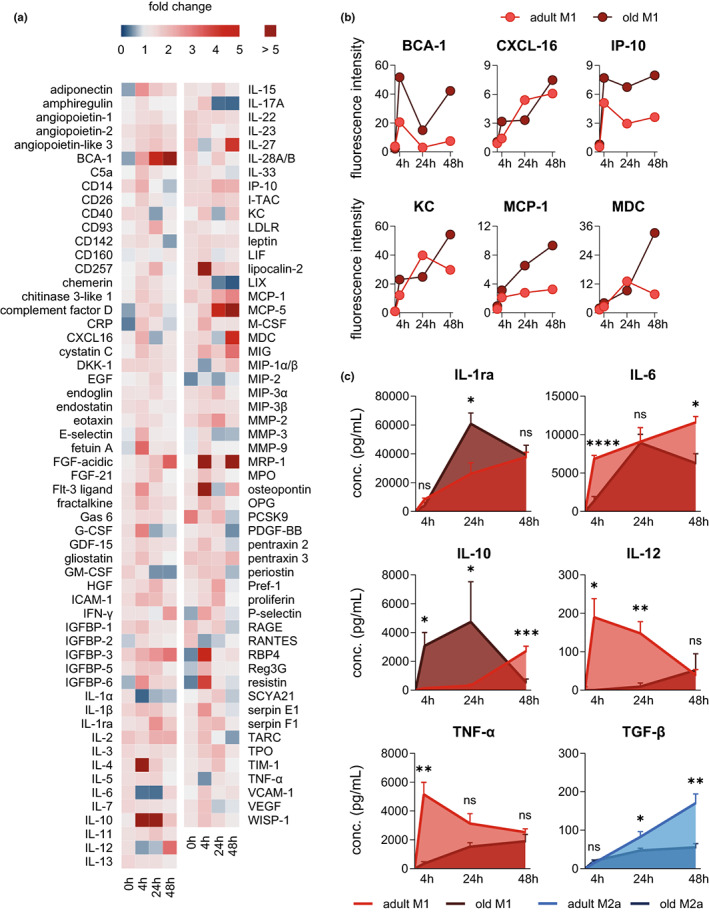
Aging leads to aberrant cytokine and chemokine release from polarized macrophages. The cytokine and chemokine secretome of adult and old PM after stimulation with LPS (100 ng/mL) and IFN‐γ (20 ng/mL was determined by means of a cytokine array (Figure S7, Table S4). (a) Heatmap showing global time‐related changes in the cytokine and chemokine secretome of old M1‐PM in comparison with adult. Fold change is implicated by color scale. (b) The temporal release is shown for selected and prominent pro‐inflammatory cytokines and chemokines secreted by M1‐PM of both age cohorts over the course of 48 h. (c) Concentration of pro‐inflammatory IL‐1ra, IL‐6, IL‐10, IL‐12, and TNF‐α in adult and old M1‐PM, and TGF‐β in adult and old M2a‐PM was measured by ELISA (*n* = 6; except 24 h [IL‐10]: *n* = 4–6). Statistics: Data are shown as (a, b) mean or (c) mean ± SEM and *p*‐values were calculated by unpaired two‐tailed Student's *t*‐test with or without Welch's correction (Table S7). *p ≤ 0.05, **p ≤ 0.01, ***p ≤ 0.001, ****p ≤ 0.0001, ns, not significant.

Next, we asked whether the divergences in the polarization process of adult and old PM translate into the abilities to secrete phenotype‐specific immunomodulatory mediators. Since cytokine and chemokine secretion from macrophages peaks at differing timepoints after LPS challenge (Rossol et al., 2011), we selected an early (4 h), intermediate (24 h), and late timepoint (48 h) during LPS/IFN‐γ‐induced M1 polarization to measure the mediator release. Aging affected the chemokine and cytokine secretome of M1‐PM in a temporal manner (Figure 5a). Most prominently, aging elevated the release of chemoattractants and activators for B cells (BCA‐1), activated T and NK cells (IP‐10), neutrophils (KC), monocytes (MCP‐1) and type 2 T helper (Th2) and cytotoxic T (Tc2) cells (MDC), particularly after 48 h of M1‐PM polarization (Figure 5a,b). Adult M1‐PM released vast amounts of pro‐inflammatory IL‐6, IL‐12, and TNF‐α already 4 h upon M1 polarization, where IL‐6 remained high and IL‐12 and TNF‐α levels dropped until 48 h (Figure 5a,c). In contrast, in old M1‐PM, the release of these cytokines was delayed. Interestingly, secretion of anti‐inflammatory IL‐1ra and IL‐10 was more rapidly induced from old PM during M1 polarization when compared to adult M1‐PM (Figure 5a,c). Note that the IL‐10 release from old M1‐PMs significantly dropped at 48 h, well below adult levels (Figure 5c). Finally, the release of anti‐inflammatory TGF‐β from M2a‐PM remained low after 24 h of polarization as consequence of aging (Figure 5c). Together, while chemokine release from M1‐PM is broadly increased by aging, the opposite holds true for pro‐inflammatory cytokines, and anti‐inflammatory ones tend to peak at odd timepoints, potentially compromising the crosstalk with other immune cells.

### 2.6 Aging shifts the metabololipidome of M2a‐PM towards pro‐inflammatory LM

**FIGURE 6:**
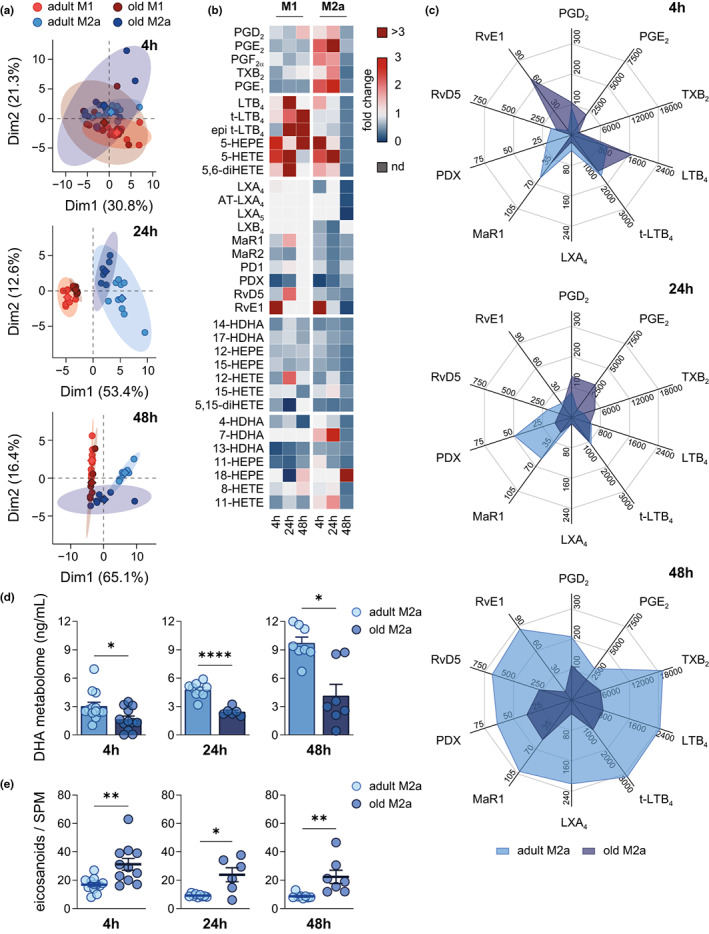
Impaired M2a macrophage polarization as consequence of aging causes a depletion of DHA‐derived SPM levels after *E. coli* infection. Polarized PM (4–48 h) were infected with pathogenic *E. coli* for 90 min at a MOI of 50 and subsequent LM levels were determined by UPL‐CM‐SMS (Table S6; 4 h: *n* = 12–13; 24 h: *n* = 6–9; 48 h: *n* = 7–8). (a) PCA of metabololipidomic profile of M1‐ and M2a‐PM from adult and old mice. (b) Heatmap showing age‐related changes in the temporal release (4–48 h) of LM from M1‐ and M2a‐PM of old mice in comparison with adult. Fold change is implicated by color scale and not detectable metabolites are marked (nd). For individual LM only detectable in one age‐group, fold change was calculated to corresponding LOD (Table S5). (c) Radar charts of selected bioactive LM released by adult and old M2a‐PM (unit: pg/mL). (d) DHA metabolome is given as sum of all metabolites of DHA released by M2a‐PM. (e) Ratio of pro‐inflammatory PGE_2_, LTB_4_, *t*‐LTB_4,_ and epi *t*‐LTB_4_ to all SPM released by M2a‐PM. Statistics: Data are shown as (b, c) mean or (d, e) mean ± SEM and *p*‐values were calculated by unpaired two‐tailed Student's *t*‐test with or without Welch's correction (Table S7). *p ≤ 0.05, **p ≤ 0.01, ****p ≤ 0.0001.

Since the M1‐ or M2‐like phenotype is tightly connected to the capacity of forming either pro‐inflammatory PG and LTs or pro‐resolving SPM, respectively (Dalli & Serhan, 2012; Werz et al., 2018), we analyzed the LM signature profiles of *E. coli*‐infected adult and old PM after M1 and M2a polarization (4–48 h). PCA revealed that already after 4 h of polarization, adult PM display slightly separated LM clusters for M1‐ and M2a‐polarized cells, but not for old PM where respective clusters widely overlap and are much broader overall (Figure 6a). The separation of LM clusters of adult M1‐ and M2a‐PM is more pronounced at 24 and 48 h, but clusters of old PM were less (24 h) or not (48 h) separated at these timepoints (Figure 6a). Since LM clusters of adult and old M1‐PM appeared rather similar, aging distinctly impacts the metabololipidome of M2a‐PM, for which the clusters were more divergent (Figure 6a).

Analysis of individual LM in M1‐ and M2a‐PM revealed temporal shifts within the metabololipidome with clear age‐related differences (Figure 6b). Already after 4 h of polarization, adult M1‐PM markedly reduced the release of LT and SPM, when compared to their naive state, while in contrast, old M1‐PM formed less PG but more LT and 5‐LOX products (Figure 6b, Figure S3a). Also, we found a significant downregulation of the entire AA metabolome in old M1‐PM after 4 h, which vanished at later timepoints (Figure S5b). At 24 h, M1‐PM of both age cohorts mainly produced PG, with reduced PGD_2_ and PGE_2_ and increased LT levels in old M1‐PM, which persisted over 48 h of polarization, except for rising PGD_2_ levels (Figure 6b, Figure S5a). Furthermore, during the whole polarization process the DHA metabolome was impaired in old M1‐PM, while the EPA metabolome differed only at 24 h between the age cohorts (Figure S5c,d).

Adult M2a‐PM shifted their metabololipidome early (4 h) from PG and LT towards SPM (LXA_4_, MaR1, PDX), while in old M2a‐PM pro‐inflammatory LM (PGE_2_, PGF_2α_, LTB_4_, t‐LTB_4_) dominated the LM profile over RvE1 as main SPM (Figure 6b,c). After 24 h, old M2a‐PM produced substantial amounts of PG, while adult M2a‐PM released various SPM (MaR1, MaR2, PD1, PDX, RvD5) (Figure 6b,c). Most strikingly, after 48 h, old M2a‐PM produced overall low amounts of LM of any class, with a striking impairment in the release of SPM (LXA_4_, AT‐LXA_4_, LXA_5_, MaR2, RvD5, RvE1) and TXB_2_ when compared to adult counterparts (Figure 6b,c). Thus, the ratio of pro‐inflammatory eicosanoids to SPM was consistently increased during polarization towards M2a as consequence of aging (Figure 6d), seemingly due to minute formation of DHA‐derived LM in old M2a‐PM (Figure 6e). Furthermore, reduced levels of EPA metabolites at both 24 and 48 h further contributed to the increased pro‐inflammatory metabololipidome in old M2a‐PM (Figure S5f). Surprisingly, adult M2a‐PM produced high levels of pro‐inflammatory LTB_4_ at the late stage of polarization (48 h) alongside an overall elevated transformation of AA (Figure 6b,c, Figure S5e). Figure S6 summarizes the temporal release of both pro‐inflammatory and pro‐resolving mediators from M1‐ and M2a‐PM and respective shifts as consequence of aging. Similar age‐associated patterns are evident among LM derived from either COX (Figure S6a) or LOX (Figure S6b), with the exception of the COX product TXB_2_ that resembles the pattern of LOX products (Figure S6a). In conclusion, aging markedly impacts the adaptation of the metabololipidome during PM polarization, particularly in M2a‐PM, which extends beyond a simple delay in SPM formation but rather implies a fundamental deficiency in LM biosynthesis.

## 3 DISCUSSION

Reduced immune response efficiencies of macrophages as consequence of aging are well recognized and known to culminate in higher susceptibility of elderly individuals to infectious diseases with unfavorable progression and outcome (Van Beek et al., 2019). However, the molecular determinants of age‐related macrophage deficiencies have not been sufficiently explored. Most studies focused solely on specific aspects of macrophage biology or functionality without detailed analysis of cellular changes that occur during macrophage aging with relevance for the immune response and inflammation (Murray et al., 2014; Prattichizzo et al., 2016). Our results show that aging compromises the development of typical M1‐ and M2a‐like macrophage phenotypes, thereby promoting a deprivation of immunologic and inflammatory pathways in old PM, which persists across ex vivo PM polarization. In particular, the temporal and context‐based adaptation of the metabololipidome in PM is considerably limited by age, which may underlie compromised homeostasis and unresolved low‐grade inflammation in elderly individuals (Doyle et al., 2018; Schädel et al., 2021).

We found markedly increased numbers of PM within in the peritoneal cavity of old mice, which displayed reduced expression of CD11b and F4/80, the most reliable discriminants for PM against other cell types within the niche (Ghosn et al., 2010). PM can be subdivided into small and large subsets, based on differences in size, expression of F4/80, and their ontogeny (Cassado Ados et al., 2015; Ghosn et al., 2010). Previous studies reported a shift in the ratio of small and large PM subtypes as consequence of inflammation or after trauma (Ghosn et al., 2010; Okabe & Medzhitov, 2014), due to monocytes being recruited from the circulation into the peritoneal niche to replace senescent or apoptotic resident macrophages (Louwe et al., 2021). Functionally, small and large PM subsets display comparable phagocytic activity, but their response to TLR4 stimulation by LPS differed significantly (Ghosn et al., 2010). This could explain the increased PM numbers in old mice and may implicate that during aging a substitution of large PM with small PM occurs through monocyte recruitment. However, no significant differences in the cell size and the phagocytic activity of PM of both age cohorts were evident, contradicting findings with mice by others (Linehan et al., 2014). Previous studies selectively addressed large PM but neglected the substantial subpopulation of small PM of old mice that contribute to the inflammatory response within the peritoneal niche (Cassado Ados et al., 2015; Ghosn et al., 2010). Notably, upon subsequent ex vivo culture for 24 h, the phagocytic capacity of adult, but not of old PM, was elevated regardless of the polarization state but vanished at 48 h. Interestingly, phagocytosis by adult PM was suppressed by the secreted mediators from LPS‐challenged old PM but not vice versa, implying that the secretome of old resident PM may detrimentally impact the phagocytic function of macrophages.

Our MS‐based proteomic profiling revealed a distinct age‐associated PM activation state, characterized by impaired inflammatory signaling pathways including mTOR, ERK/MAPK, NRF2, TREM1 and a rise in apoptosis signaling. Furthermore, canonical pathways analysis showed reduced sirtuin signaling in old PM, implying a more quiescent state and impaired self‐renewal (Imperatore et al., 2017). Also, the protein levels of COX‐1, cPGES, and FLAP, which are key enzymes in the biosynthesis of pro‐inflammatory PG and LT (Radmark et al., 2015; Smith et al., 2011), were decreased in old PM, along with considerably lower PGE_2_ and LTB_4_ formation upon infection with pathogenic *E. coli*. Note that in old PM also formation of SPM (i.e., PD, RvD5, and MaR1) was impaired, suggesting potential deficits in immune response and tissue repair (Markworth et al., 2021). However, there was no dominance of pro‐inflammatory PG and LT over SPM, which has been suggested as key feature of inflammaging (Arnardottir et al., 2014; Minhas et al., 2021). Side‐by‐side analysis of PM with SM and BMDM showed that age‐related suppression of PG and TX after infection with pathogenic *E. coli* is a common feature among macrophages from different niches.

Previous studies suggest a rather pro‐inflammatory M1‐like phenotype for tissue‐resident macrophages as consequence of aging (Becker et al., 2018; Van Beek et al., 2019). Our data from proteomic and metabololipidomic profiling revealed a distinct age‐related phenotype for PM and, at least in terms of LM profile, also for SM and BMDM, which cannot be readily assigned to either an inflammatory M1‐like or pro‐resolving M2a‐like phenotype. There is a general consensus that the classical categorization into the extreme phenotypes M1 and M2 is difficult to apply to macrophage populations in vivo, where a mixture of different activation states is common (Murray et al., 2014). In our study, several proteins and signaling pathways characteristic for M1 (e.g., CD86, HO‐1, iNOS, and SOD‐2) and for M2a (CD200R, TLR8, YM1, DMP‐4) were downregulated in old PM. Yet, other markers for M1 (CD54, GSTA3, IGF‐2, mTOR) or M2a (CD206, IRF‐8, SLC1A5, PFKFB4) were elevated as consequence of aging. Therefore, our results underline the necessity to expand the classification of macrophages beyond the simple M1 and M2 dichotomy and to consider broader sets of phenotypic protein markers and immunomodulatory mediators, especially LM, to adequately attribute inflammatory or pro‐resolving characteristics (Dalli & Serhan, 2012; Ley, 2017; Murray et al., 2014; Werz et al., 2018).

A phenotypic switch from M1‐ to M2a‐PM is crucial for resolving inflammation, tissue repair, and regeneration to eventually return to homeostasis (Serhan & Savill, 2005; Watanabe et al., 2019). Although proteomic analysis after ex vivo polarization revealed marked alterations in the acquired M1 and M2a phenotype of both PM age cohorts, the PCA revealed separated clusters only for adult M1‐ and M2a‐PM, while those of old PM overlap, albeit their morphology and functionalities should differ extensively (Wculek et al., 2021; Werz et al., 2018). Interestingly, aging affects M1 and M2a polarization of PM and subsequent adaptation of signaling pathways in an opposite manner. Along this line, integrin signaling, which is critical for macrophage effector functions at the site of infection and required for host defense (Brown, 2003), is markedly decreased in M1—but elevated in M2a‐PM. Moreover, old PM may fail to adapt vast parts of their metabolism and intra‐ and intercellular signaling during M1 polarization, as proteins involved in glycolysis, glycogen degradation, and fatty acid oxidation as well as NRF‐mediated oxidative stress response, leukocyte extravasation, chemokine signaling, and inflammation‐related pathways (Orecchioni et al., 2019) were downregulated. Recently, aging was found to suppress myeloid cell bioenergetics and thereby drives pro‐inflammatory responses leading to tissue malfunction like cognitive decline (Minhas et al., 2021). On the contrary, old M2a‐PM displayed markedly elevated levels of inflammatory markers such as IGF‐2, mTOR, LTF, and PRDX‐1 as well as increased signaling pathways like ILK and integrin signaling, production of NO and ROS, and TNFR signaling that are actually associated with the M1 phenotype (Orecchioni et al., 2019). At the same time, proteins that are associated with the M2a phenotype in adult PM (i.e., ARG‐1, CD200R, IRF‐8, and SLC1A5) (Orecchioni et al., 2019) are attenuated by aging. Therefore, it appears that aging compromises the degree to which PM can adapt functionally opposing polarization states.

Recognition, uptake and presentation of PAMPs are major tasks of macrophages (Greene et al., 2022). During polarization of adult PM, the phagocytic activity peaked after 24 h, with a higher activity in M1‐ versus M2a‐PM. Aging abolished both the time‐ and polarization‐dependency of the phagocytic process, which is in line with changes in phagocytosis of aged macrophages connected to a loss of diurnally rhythmic innate immune responses (Blacher et al., 2022). Upon recognition of PAMPs, macrophages orchestrate the immune response through the release of chemoattractant and immunomodulatory mediators (Arango Duque & Descoteaux, 2014; Davies et al., 2013). Tailored temporal release of different chemokines, cytokines, and LM is crucial for mounting the host immune response (Rossol et al., 2011; Serhan & Savill, 2005). Our secretome analysis revealed elevated release of chemoattractants and activators of cells in innate and adaptive immunity from old M1‐PM, especially at later timepoints (i.e., 48 h). These data concur with the age‐related increased recruitment and persistence of immune cells within various niches like the adipose tissue, liver, lung, peritoneum, and spleen (Almanzar et al., 2020; Mogilenko et al., 2021). Along these lines, aging caused delayed and impaired release of the pro‐inflammatory cytokines IL‐6, IL‐12, and TNF‐α but accelerated and elevated secretion of anti‐inflammatory IL‐1ra and IL‐10 during M1 polarization. Such aberrations may lead to miscommunication between PM and other immune cells at the site of infection (Davies et al., 2013).

Transition from inflammation to resolution is accompanied by a LM class switch from pro‐inflammatory PG and LT to pro‐resolving SPM, which is directly connected to the polarization state of macrophages at sites of inflammation (Serhan, 2014; Serhan et al., 2015; Werz et al., 2018). Aging affected LM signature profiles in M1‐PM mainly by lowering the formation of the major metabolite PGE_2_ across the polarization process, which can be explained by decreased COX‐1 and cPGES levels in old M1‐PM at early timepoints, while attenuation of mPGES‐1 expression may limit PGE_2_ formation at the later polarization stage. Polarization of adult PM towards M2a progressively shifted the LM profile from PG and LT to SPM formation, which was not the case for old M2a‐PM. Consequently, the ratio of pro‐inflammatory eicosanoids to SPM markedly increased in old M2a‐PM during polarization, mainly caused by impaired DHA metabolism. Our data thus complement previous findings, where aging delayed resolution in a model of self‐resolving peritonitis (Arnardottir et al., 2014) with mechanistic insights into the role that PM play.

Conclusively, aging has a tremendous impact on the activation and functionality of PM resulting in aberrant macrophage phenotypes outside of the simplified M1 and M2 dichotomy. Our findings fit with the general observation that elderly individuals are prone to develop unresolved, chronic inflammation and are at higher risk to die from infections, obviously apparent in the COVID‐19 pandemic (Fulop et al., 2021). Yet, our data challenge the dogma of increased pro‐inflammatory macrophage pre‐activation due to aging and instead reveal maladaptive properties in the plasticity and immunological competence of macrophages.

## 4 EXPERIMENTAL PROCEDURES

### 4.1 Mice

Adult (4–6 months) and old (24 months) male C57BL/6JRj mice were obtained from Janvier Laboratories or bred internally at the animal facility of the Leibnitz Institute on Aging – Fritz Lipmann Institute (FLI). All animals were house under standardized conditions in a specific pathogen‐free facility with a 12 h light/dark cycle either within the FLI or the Bioinstrumentezentrum. Laboratory animals were killed, and samples were harvested in accordance with the animal welfare laws of Germany and the European Union.

### 4.2 Isolation, culture, and polarization of peritoneal macrophages

Mice from both age cohorts were euthanized with carbon‐dioxide inhalation in accordance with standard operation procedures at the Leibnitz Institute on Aging – FLI or the Bioinstrumentezentrum. Immediately after death, 10 mL of ice‐cold RPMI 1640 medium (Sigma Aldrich, R8758) was injected into the peritoneal cavity, and after gentle agitation, the peritoneal lavage was recovered. The cell suspension was transferred into 15 mL conical flasks and temporarily stored on ice. After centrifugation (4°C, 10 min, 2000 *g*), the supernatant was discarded and cells were resuspended in 1 mL of macrophage medium (RPMI 1640, 10% heat‐inactivated fetal calf serum [FCS, Sigma Aldrich, F75249], 2 mM L‐glutamine [Merck; G7513], 100 U/mL penicillin, and 100 μg/mL streptomycin [Sigma Aldrich, P0781]). Cell number, viability, size, and circularity were assessed with a Vi‐CELL XR system (Beckman Coulter). Cells were seeded at a density of 10^6^ cells per mL in macrophage medium. After 2 h of adherence, PM were aspirated and processed for further analysis. For polarization experiments, PM were incubated with LPS (100 ng/mL; Sigma Aldrich; 13636230) and murine IFN‐γ (20 ng/mL; Peprotech; 315‐05) or murine IL‐4 (20 ng/mL; Peprotech, 214‐14) for either 4, 24, or 48 h to induce M1 or M2a phenotypes, respectively. Supernatants were collected at given timepoints and stored at −80°C for further analysis.

### 4.3 Surface marker analysis by flow cytometry

After cultivation/polarization, macrophages were detached with flow cytometry buffer (PBS pH 7.4, SERVA; 47302.03), 0.5% (w/v) bovine serum albumin (AppliChem; A1391), 2 mM EDTA (AppliChem; A2937), 0.1% sodium azide (Merck, S2002) with additional 0.4% (w/v) lidocaine (Sigma Aldrich, L7757) and transferred into to a fresh tube. After centrifugation (20°C, 10 min, 400 *g*), the supernatant was discarded and PM were stained with Zombie Aqua™ (BioLegend; 423101) before blocking non‐specific antibody binding using rabbit serum (Fisher Scientific; 11829230). Subsequently, PM were stained in flow cytometry buffer with the following antibodies: FITC anti‐mouse CD11b (BioLegend; 101206), APC anti‐mouse F4/80 (BioLegend; 123116), PE/Cyanine7 anti‐mouse CD54 (BioLegend; 116122), PerCP anti‐mouse CD86 (BioLegend; 400530), PE anti‐mouse CD200R (BioLegend; 1239089), and Brilliant Violet 421™ anti‐mouse CD206 (BioLegend; 141717). Surface markers were detected using BD LSR Fortessa (BD Biosciences) according to an established protocol. Data were analyzed with FlowJo 10.8 software (BD Biosciences).

### 4.4 Phagocytosis assay

After initial seeding, PM were aspirated, supplied with fresh macrophage medium (RPMI 1640, 10% heat‐inactivated FCS, 2 mM L‐glutamine, 100 U/mL penicillin, and 100 μg/mL streptomycin) and incubated with 5 μL of green‐fluorescent *E. coli* particles (abcam, ab235900) per well (37°C, 2 h, 5% CO_2_). After incubation, cells were carefully aspirated and washed repeatedly, and finally, 500 μL of assay buffer was added. Fluorescence was measured with a Novostar system (BMG Labtech), and relative phagocytosis activity was calculated through a standard curve of different concentrations of green fluorescent *E. coli* particles in assay buffer.

### 4.5 Cell lysis and digestion for proteome analysis

Peritoneal macrophages were lysed in Tris‐buffered saline (TBS; 200 mM Tris base, 0.15 M sodium chloride, pH 7.4) including 1% (v/v) Nonidet‐P40, 1 mM sodium orthovanadate, 10 mM sodium fluoride, 5 mM sodium pyrophosphate, 25 mM β‐glycerophosphate, 5 mM EDTA, 25 μM leupeptin, 3 μM soybean trypsin inhibitor, and 1 mM phenylmethanesulfonyl fluoride. Reduction and alkylation of cysteines, protein precipitation and digestion, and peptide preparation for UPLC‐MS‐MS analysis were performed as described in detail in the Appendix S1.

### 4.6 Proteome analysis by UPLC‐MS‐MS

For spectral library generation, peptides were separated using a nanoAcquity UPLC system (Waters) fitted with a trapping (nanoAcquity Symmetry C18, 5 μm, 180 μm × 20 mm) and an analytical column (nanoAcquity BEH C18, 1.7 μm, 75 μm × 250 mm). Samples (~1 μg) were loaded with a constant flow of water with 0.1% formic acid (solvent A) at 5 μL/min onto the trapping column. Trapping time was 6 min, and peptides were eluted via a nonlinear gradient from 1% to 62.5% acetonitrile with 0.1% formic acid (solvent B) in 131 min. Total runtime was 145 min, including clean‐up and column re‐equilibration. Peptides were introduced into the mass spectrometer (Thermo Q‐Exactive HFX; Thermo Fisher Scientific) via a Pico‐Tip Emitter 360 μm OD × 20 μm ID; 10 μm tip (New Objective), and a spray voltage of 2.2 kV was applied. The RF ion funnel was set to 40%.

The conditions for data dependent acquisition (DDA) and data independent acquisition (DIA) are reported in detail in the Supporting Information.

### 4.7 Proteome data analysis

Acquired data were processed using Spectronaut™ Professional 13 (Biognosys AG). For library creation, the DDA and DIA raw files were searched with Pulsar (Biognosys AG) against the mouse UniProt database (Mus musculus, entry only, release 2016_01) with a list of common contaminants appended. For library generation, default BGS factory settings were used. The library contained 87,176 precursors, corresponding to 4746 protein groups using Spectronaut™ protein inference. DIA data were analyzed and searched against the specific spectral libraries using Spectronaut™. Relative quantification was performed in Spectronaut™ for each pairwise comparison using the replicate samples from each condition. The data were searched with the following modifications: Carbamidomethyl (C) (Fixed), and Oxidation (M) and Acetyl (Protein N‐term) (Variable). A maximum of 2 missed cleavages for trypsin were allowed. The identifications were filtered to satisfy false discovery rate (FDR) of 1% on peptide and protein level. Protein quantification was performed in Spectronaut™ using default settings except: Proteotypicity Filter = Only Protein Group Specific; Major Group Quantity = Median peptide quantity; Major Group Top N = OFF; Minor Group Quantity = Median precursor quantity; Minor Group Top N = OFF; Normalization Strategy = Local Normalization; Normalize on = Median; Row Selection = Identified in at least 1 run (Sparse). Differential abundance testing was performed in Spectronaut™ using a paired t‐test between replicates. *p*‐Values were corrected for multiple testing with the method described by Storey (Storey, 2002). Data candidate tables and protein quantity data reports (Table S1) were exported from Spectronaut™ and used for visualization and statistical analysis. Candidate tables from Spectronaut™ were used as input for canonical pathways using Ingenuity Pathway Analysis software (version 01‐14; QIAGEN Inc.). A cutoff for significance of *q* < 0.05 and absolute fold change (log_2_) > 0.58 was used, respectively.

### 4.8 Cytokine and chemokine profiling

Cytokine levels in supernatants from PM of adult and old mice were measured using either an enzyme‐linked immunosorbent assay (ELISA) or by cytokine arrays. All materials were purchased from R&D systems (IL‐1beta/IL‐1F2: DY401‐05; IL‐1ra/IL‐1F3: DY480; IL‐6: DY406; IL‐10: DY417 05; IL‐12 p70: DY419 05; TGF‐beta‐1: DY1679‐05; TNF‐alpha: DY410‐05; Proteome Mouse XL Cytokine Array Kit: ARY028). Assays were performed according to the manufacturer instructions for the ELISAs. For cytokine arrays, samples from all biological replicates of an experimental group were pooled and used for the assay. Detection of the array spots was done with fluorescent‐labelled Streptavidin‐HRP (LI‐COR Biosciences, 926‐32230), and arrays were analyzed using an Odyssey infrared imager (LI‐COR Biosciences). Densitometric analysis of array spots and background subtraction was performed using manufacturers software (Table S4).

### 4.9 Infection of PM with pathogenic *E. coli* for LM formation

Pathogenic *E. coli* (serotype O6:K2:H1) were grown overnight in nutrient broth (NB) medium under constant shaking at 37°C. Bacterial culture was diluted to OD_600nm_ of 0.1 and incubated for another 3–4 h until it reached an OD_600nm_ of 1.0. Bacteria were pelleted by centrifugation (20°C, 5 min, 3350 *g*) and resuspended in PBS pH 7.4 containing 1 mM CaCl_2_ (AppliChem, C3306). For infection, 1 × 10^6^ PM were kept in PBS pH 7.4 with 1 mM CaCl_2_ and incubated with prepared *E. coli* suspensions (37°C, 5% CO_2_, 90 min) at a multiplicity of infection (MOI) of 50.

### 4.10 Sample preparation and metabololipidomic profiling by UPLC‐tandem mass spectrometry

To the supernatants from co‐incubations of PM and *E. coli*, deuterium‐labelled LM standards were added, and samples were prepared by solid phase extraction for LM profiling by UPLC‐MS‐MS as described in detail in the Supporting Information.

Profiling of bioactive LM, their mono‐hydroxylated precursors and corresponding PUFAs was performed by UPLC‐MS‐MS following an established protocol (Werner et al., 2019) and presented in detail in the Supporting Information. Retention time of each analyte was compared to an external standard, and linear standard curves with *r* ^2^ values of 0.98–0.99 were obtained for quantification. Further, limit of detection (LOD) was established for each analyte (Table S5). Recovery rates of internal deuterium‐labelled LM standards were determined for each sample and used to account for deviations between replicates as results of sample preparation and handling. Values are given as pg of LM per mL of sample, if not stated otherwise (Table S6).

### 4.11 Data handling and statistical analysis

Sample size for all performed experiments was based on previous experiments to ensure adequate statistical power, and was repeated on different days to eliminate bias. Results, if not stated otherwise, are given as mean including the standard error of the mean (SEM) of a given number of experiments (*n*) with samples from different donors. Data analysis and visualization were performed using R Studio (version 1.4) or GraphPad Prism (version 9.2.0). Principal component analysis (PCA) was implemented into R code by using the R packages FactoMineR (https://cran.r‐project.org/web/packages/FactoMineR/index.html) and factoextra (https://cran.r‐project.org/web/packages/factoextra/index.html) and applied to metabololipidomic and proteomic data. To identify outliers within the data sets, a regression and outlier removal (ROUT) test within Prism software was used (Motulsky & Brown, 2006) with a *q*‐value set to 0.1, and detected outliers were excluded from further analysis. Data distribution was assessed by Shapiro–Wilk test to identify normal or log‐normal distribution of data. Log‐normal distributed data were hence log‐transformed for statistical analysis. If not stated otherwise, comparison of two‐groups was assessed by unpaired two‐tailed Student's *t*‐test and Welch Correction employed, if F‐test yielded unequal standard deviations (*p* < 0.05). For comparison of more than two datasets, one‐way ANOVA for multiple comparisons with Šídák post‐hoc test was used or Brown‐Forsythe and Welch ANOVA with Dunnett T3 post‐hoc test, if both tests proved significant differences among means (*p* < 0.05). Results from statistical comparisons can be reviewed in Table S7. Statistical significance was assumed for comparisons with *p* < 0.05 and is indicated as: *p ≤ 0.05, **p ≤ 0.01, ***p ≤ 0.001, ****p ≤ 0.0001, ns, not significant. For proteome data, proteins with a *q*‐value below 0.05 and absolute log2‐fold change above 0.58 were considered as significantly affected, unless otherwise stated. Displayed q‐values were calculated by Spectronaut™ and indicated as *q < 0.05, **q < 0.01, ***q < 0.001, ns, not significant, unless otherwise stated.

## AUTHOR CONTRIBUTIONS

PS and AC performed the experiments and evaluated the data from the metabololipidomic profiling, flow cytometry, phagocytosis, and cytokine release. PS and NG performed the experiments and evaluated the data from the proteomic profiling. PS, AC, and NG visualized the findings and performed statistical analysis. PS, OW, and AO designed the study. PS and OW drafted the work and AC, NG, IDJ, and AO substantively revised it. All authors read and approved the final manuscript.

## FUNDING INFORMATION

The authors gratefully acknowledge support from the mouse facilities of the FLI and BIZ as well as the Proteomics Core Facility at FLI. The study was supported by the Free State of Thuringia, ProExcellence Initiative 2 (“RegenerAging”), the Deutsche Forschungsgemeinschaft (DFG, German Research Foundation, SFB 1127 ChemBioSys, project number 239748522; Germany's Excellence Strategy – EXC 2051 Balance of the Microverse – Project‐ID 390713860), and the Carl Zeiss Foundation (IMPULS). The FLI and HKI are members of the Leibniz Association and are financially supported by the Federal Government of Germany and the State of Thuringia. Schemes and graphics were created with BioRender.com.

## CONFLICT OF INTEREST STATEMENT

The authors declare no competing interests.

## Supporting information


Appendix S1.

Table S1.

Table S2.

Table S3.

Table S4.

Table S5.

Table S6.

Table S7.

## Data Availability

The mass spectrometry proteomics data have been deposited to the ProteomeXchange Consortium via the PRIDE partner repository (Perez‐Riverol et al., 2019) with the dataset identifier PXD027357.
