# Supplementary material for: Metabololipidomic and proteomic profiling reveals aberrant macrophage activation and interrelated immunomodulatory mediator release during aging
Source: Aging Cell. 2023 Apr 26;22(7):e13856. doi: 10.1111/acel.13856 (PMC10352559; doi:10.1111/acel.13856)
Supplement: Supplementary file 1 — Appendix S1. [file ACEL-22-e13856-s008.pdf]

Supplement: Metabololipidomic and proteomic profiling reveals aberrant macrophage activation and interrelated immunomodulatory mediator release during aging

Patrick Schädel[1], Anna Czapka[1,2], Nadja Gebert[3], Ilse Denise Jacobsen[2,4], Alessandro Ori[3,*] and Oliver Werz[1,*]

[1] Department of Pharmaceutical/Medicinal Chemistry, Institute of Pharmacy, Friedrich-Schiller University, Jena, Germany

[2] Leibniz Institute for Natural Product Research and Infection Biology - Hans Knoell Institute (HKI), Jena, Germany

[3] Leibniz Institute on Aging – Fritz Lipmann Institute (FLI), Jena, Germany

[4] Institute of Microbiology, Friedrich-Schiller University, Jena, Germany

[*] corresponding authors: Alessandro Ori, Oliver Werz

mailing addresses: Department of Pharmaceutical/Medicinal Chemistry, Institute of Pharmacy, Friedrich Schiller University Jena, Philosophenweg 14, 07743 Jena, Germany

email address: oliver.werz@uni-jena.de

telephone number: +49 3641 949801

Leibniz Institute on Aging – Fritz Lipmann Institute (FLI), Jena, Germany

email address: alessandro.ori@leibniz-fli.de

telephone number: +49 3641 65-6808

Supplementary methods

Isolation, magnetic separation and culture of splenic macrophages (SM)

Spleens from mice of both age cohorts were transferred into ice-cold PBS (pH 7.4, SERVA; 47302.03) and kept at 4 °C until further processing. The tissue was dissociated using a commercially available kit (Miltenyi Biotec; 130-095-926) in combination with a gentleMACS™ Octo Dissociator (Miltenyi Biotec; 130-096-427) according to manufacturer's instructions. The single cell suspension was purified with help of a debris removal kit (Miltenyi Biotec; 130-109-398) and red blood cells were removed through hypotonic lysis (G-Biosciences; 786-672). Cells were stained with magnetically labelled anti-F4/80 MicroBeads (Miltenyi Biotec; 130-110-443) and separated on a MS column (Miltenyi Biotec; 130-122-727) via OctoMACS™ separator (Miltenyi Biotec, 130-042-108). Cell number, viability, size, and circularity of F4/80-positive SM was assessed with a Vi-CELL XR system (Beckman Coulter). SM were seeded at a density of 10^6 cells per mL in macrophage medium and kept at 37 °C and 5% CO_2 until further analysis.

Isolation, differentiation and culture of bone marrow-derived macrophages (BMDM)

Immediately after euthanisa, the hind legs of both adult and old mice were separated from the hip juncture and adjacent muscle tissue removed. Both tibia and femur were collected in ice-cold PBS (pH 7.4) and kept at 4 °C until further processing. Bones were later cut at both ends and the bone marrow flushed with a 25G needle into a conical tube. After centrifugation (4 °C, 10 min, 300 *g*) red blood cells were removed through hypotonic lysis (G-Biosciences; 786-672). Cell suspension was centrifuged (4 °C, 10 min, 300 *g*), resuspended in macrophage medium and passed through a 70 µm strainer. Cell number, viability, size, and circularity of bone marrow cells was assessed with a Vi-CELL XR system (Beckman Coulter). 15 x 10^6 cells were resuspended in 10 mL macrophage medium with 25 ng/mL murine macrophage colony-stimulating factor (M-CSF, PeproTech; 315-02) and cultured in 75 cm^2 cell culture flask (Greiner Bio-One; 658170) at 37 °C and 5% CO_2. After 3 and 6 d, supernatant was removed and cells supplied with fresh medium. After 9 d of culture, cells were detached (PBS pH 7.4

with 5 mM EDTA (AppliChem; A2937)) and cell parameters assessed before re-seeding at a density of 10^6 cells per mL in macrophage medium. Bone marrow-derived macrophages (BMDM) were kept at 37 °C and 5% CO_2 until further analysis.

Surface marker analysis of macrophages from different immunological niches by flow cytometry

Macrophages (i.e., PM, SM and BMDM) were resuspended in flow cytometry (FC) buffer (PBS pH 7.4, SERVA; 47302.03), 0.5% (w/v) bovine serum albumin (AppliChem; A1391), 2 mM EDTA (AppliChem; A2937), 0.1% sodium azide (Merck, S2002)) and seeded in a 96-well plate (Boettger, 05-021-0100) at a density of 10^5 cells per 100 µL. After centrifugation (4 °C, 5 min, 300 *g*), the supernatant was discarded and macrophages were stained with Zombie Aqua™ (BioLegend; 423101). Supernatant was aspirated and cells resuspended in FC buffer before blocking non-specific antibody binding using a mouse-specific FcR blocking reagent (Miltenyi; 130-092-575). Subsequently, macrophages were stained with the following antibodies: FITC anti-mouse CD11b (BioLegend; 101206) or FITC anti-mouse CD45 (Invitrogen; 11-0451-85); APC anti-mouse F4/80 (BioLegend; 123116), PE/Cyanine7 anti-mouse CD54 (BioLegend; 116122), PerCP anti-mouse CD86 (BioLegend; 400530) and PE anti-mouse CD200R (BioLegend; 1239089) at 4 °C for 20 min in the dark. Staining was stopped with 150 µL of FC buffer, supernatant discarded, and cells were washed twice with FC buffer. Finally, macrophages were resuspended in 200 µL of FC buffer for measurement via a BD FACSVerse™ (BD Biosciences). Additionally, compensation of fluorescent spillover within the used FC panels was performed by employing OneComp eBeads™ (ThermoFisher Scientific; 01-1111-41) and Ultracomp eBeads™ Plus compensation beads (ThermoFisher Scientific, 01-3333-42). Data were analysed with FlowJo 10.8 software (BD Biosciences).

Cell lysis and digestion for proteome analysis

PM were lysed in Tris-buffered saline (TBS; 200 mM Tris base (Carl Roth; AE15.3), 0.15 M sodium chloride (Carl Roth; HN00.3), pH 7.4) including 1% (v/v) Nonident-P40 (AppliChem;

A1694), 1 mM sodium orthovanadate (AppliChem; A2196), 10 mM sodium fluoride (AppliChem; A3904), 5 mM sodium pyrophosphate (Sigma Aldrich, S8282), 25 mM β-glycerophosphate (Sigma Aldrich, G9422), 5 mM EDTA (AppliChem; A2937), 25 µM leupeptin (Sigma-Aldrich; L2884), 3 µM soybean trypsin inhibitor (Sigma Aldrich; T9128) and 1 mM phenylmethanesulfonyl fluoride (Sigma Aldrich; P7626). Lysates were clarified by centrifugation (4 °C, 10 min, 21,100 *g*) and transferred into fresh tubes. For reduction and alkylation of cysteines, lysates were incubated with 10 mM dithiothreitol (DTT; Carl Roth; 6908.3) at 37 °C for 30 min and subsequently treated with 15 mM iodoacetamide (Sigma #I1148) for 30 min in the dark. For protein precipitation, samples were treated with 8-fold volume of acetone (Biosolve, 010306) and kept at -20 °C overnight. After centrifugation (4 °C, 1 h, 20,800 *g*) the supernatant was removed and precipitates were washed twice with 500 µL of 80% (v/v) acetone in Milli-Q water. Pellets were air-dried and resuspended in digestion buffer (3 M urea (Sigma Aldrich; U6504) in 100 mM HEPES (Sigma Aldrich; H3375), pH 8.0). Samples were subjected to 3 cycles (1 min on, 30 s off) of sonication in a Bioruptor system (Diagenode) with high intensity at 20 °C. Lysyl endopeptidase (Wako Chemicals, 125-05061) was added to the samples at a ratio of 1:100 (w/w) and proteins were digested under gentle agitation (37 °C, 4 h, 300 rpm). Afterwards, samples were diluted in equal amount of Milli-Q water and further digested with trypsin (Promega; 5111) at a ratio of 1:100 (w/w) under gentle agitation (37 °C, 16 h, 300 rpm). Digested peptide solutions were acidified with 10% (v/v) trifluoroacetic acid (Biosolve; 202378) and desalted with Waters Oasis® HLB µElution Plate 30 µm (Waters; 186001828BA). During this process, columns were repeatedly (3x) conditioned with 100 µL of 80% (v/v) acetonitrile (Biosolve; 012078) and repeatedly (3x) equilibrated with 100 µL of 0.05% (v/v) formic acid (Biosolve; 069178) in Milli-Q water. Samples were loaded, washed 3 times with 100 µL 0.05% (v/v) formic acid in Milli-Q water, and eluted into PCR tubes with 50 µL of 80% (v/v) acetonitrile. Eluates were dried with a speed vacuum centrifuge and dissolved in 5% (v/v) acetonitrile and 0.1% (v/v) formic acid to a peptide concentration of 1 µg/µL. Finally, 20 µL of sample were transferred into a glass vial with inserts and 0.25 µL of

hyper reaction monitoring (HRM) peptides (Biognosys; 42896) were spiked into each sample prior to analysis.

Proteome analysis by UPLC-MS-MS

For spectral library generation, peptides were separated using a nanoAcquity UPLC system (Waters) fitted with a trapping (nanoAcquity Symmetry C18, 5 µm, 180 µm x 20 mm) and an analytical column (nanoAcquity BEH C18, 1.7 µm, 75 µm x 250 mm). Samples (~1 µg) were loaded with a constant flow of water with 0.1% formic acid (solvent A) at 5 µL/min onto the trapping column. Trapping time was 6 min and peptides were eluted via a non-linear gradient from 1% to 62.5% acetonitrile with 0.1% formic acid (solvent B) in 131 min. Total runtime was 145 min, including clean-up and column re-equilibration. Peptides were introduced into the mass spectrometer (Thermo Q-Exactive HFX; Thermo Fisher Scientific) via a Pico-Tip Emitter 360 µm OD x 20 µm ID; 10 µm tip (New Objective) and a spray voltage of 2.2 kV was applied. The RF ion funnel was set to 40%.

The conditions for data dependent acquisition (DDA) were as follows: Full scan mass spectra with a mass range of 350-1650 m/z were acquired in the Orbitrap with resolution of 60,000 Full width at half-maximum (FWHM). The filling time was set at a maximum of 20 ms with a maximum target capacity of the C-trap (AGC target) of $3x10^6$ ions. A Top15 method was employed to select precursor ions from the full scan mass spectra for fragmentation, quadrupole isolation (1.6 m/z) and measurement in the Orbitrap (resolution: 15,000 FWHM, fixed first mass: 120 m/z). The fragmentation was performed after accumulation of $2x10^5$ ions or after filling time of 25 ms for each precursor ion (whichever occurred first). Only multiply charged (2^+-7^+) precursor ions were selected for tandem mass spectrometry. Dynamic exclusion was employed with maximum retention period of 20 s. Isotopes were excluded.

The conditions for data independent acquisition (DIA) were modified as follows: Full scan mass spectra with a mass range of 350-1650 m/z were acquired in profile mode in the Orbitrap with resolution of 120,000 FHWM. The filling time was set at a maximum of 60 ms with limitation of $3x10^6$ ions. DIA scans were acquired with 40 mass window segments of differing widths across

the MS1 mass range. Higher energy C trap dissociation (HCD) fragmentation (stepped normalised collision energy: 25.5, 27, 30%) was applied and tandem mass spectra were acquired with a resolution of 30,000 FHWM with a fixed first mass of 200 m/z after accumulation of $3x10^6$ ions or after filling time of 40 ms (whichever occurred first). Data were acquired in profile mode. For data acquisition and processing Tune version 2.9 and Xcalibur 4.1 were employed.

Sample preparation for metabololipidomic profiling

Supernatants from co-incubations of PM and *E. coli* were transferred to glass vials with a 2-fold amount of ice-cold methanol (fisher chemical; 10653963) containing deuterium-labelled LM standards, which include 200 nM d8-5S-hydroxyeicosatetraenoic acid (HETE) (Cayman Chemical; 334230), d4-leukotriene B_4 (Cayman Chemical; 320110), d5-lipoxin A_4 (Cayman Chemical; 24936), d5-resolvin D2 (Cayman Chemical; 11184), d4-prostaglandin E_2 (Cayman Chemical; 10007273) and 10 µM d8-arachidonic acid (Cayman Chemical; 390010). Samples were stored overnight at -20 °C to ensure protein precipitation before further processing. After centrifugation (4 °C, 10 min, 1200 *g*), the supernatant was transferred to a fresh vial and acidified by addition of 9 mL Milli-Q water (pH 3.5) for solid phase extraction (SPE). SPE cartridges (Waters, WAT043395) were first washed with 6 mL ice-cold methanol and equilibrated with 2 mL of Milli-Q water before sample loading. Immediately afterwards, loaded cartridges were washed with 6 mL of Milli-Q water to return to neutral pH and with 6 mL of *n*-hexane (fisher chemical; 10703611). Finally, LM and remaining fatty acids were eluted with 6 mL of methyl formate (fisher chemical; 414340025) into glass vials and samples were evaporated until dryness with a moderate stream of nitrogen before resuspension in 100 µL of an equal mixture of methanol and water (VWR Chemicals, 83645320).

Supplementary figures

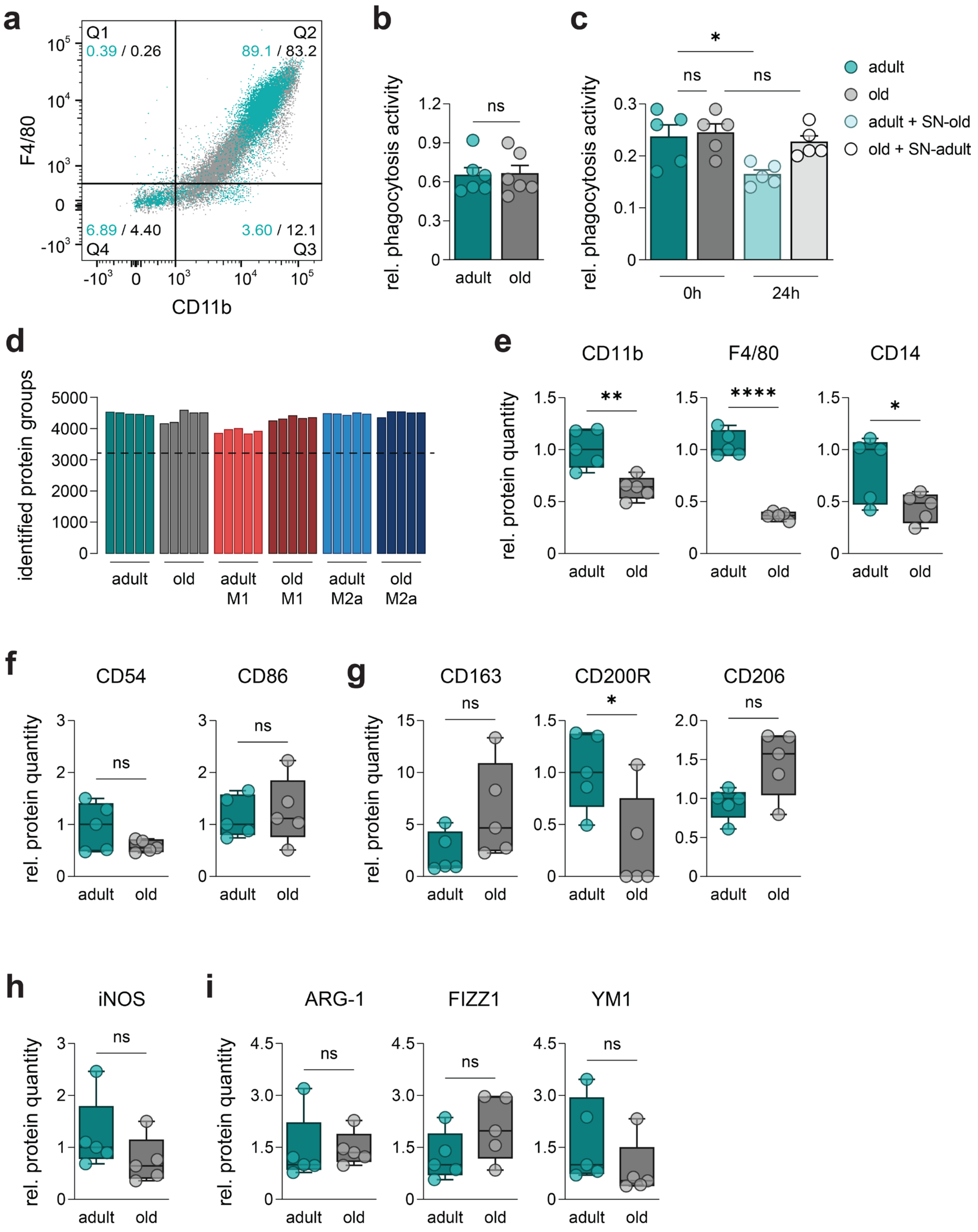

Figure S1: Aging affects the phenotype of resident peritoneal macrophages.

a) Expression of surface markers CD11b and F4/80 on PM from adult (turquoise) and old mice (grey) was measured by flow cytometry (n = 9-10). Results are shown in a representative overlaid dot plot indicating percental distribution of populations. **b)** PM from adult and old mice were incubated with fluorescent-labelled *E. coli* particles for 2 h and phagocytic activity was determined by measuring fluorescence after uptake (n = 6). **c)** PM of adult and old mice were stimulated with LPS (100 ng/mL) for 18 h and afterwards supernatant (SN) of both age cohorts was swapped and cells were incubated for another 6 h. Phagocytic activity was assessed for naive adult and old PM before (0 h) and after (24 h) challenging with SN from the other age group (n = 5). **d)** Number of identified protein groups in naive and polarized PM from adult (4-6 months) and old mice (> 24 months) determined by DIA mass spectrometry (n = 5). Dotted line indicates the number of proteins all cohorts have in common. Levels of specific surface markers for **e)** PM (CD11b, F4/80 and CD14), **f)** M1 (CD54, CD86) and **g)** M2a macrophages (CD200R, CD206) were determined in adult and old PM by DIA mass spectrometry (n = 5). Levels of intracellular protein markers for **h)** M1 (iNOS) and **i)** M2a (ARG-1, FIZZ1, YM1 and YM2) macrophages were determined in PM of adult and old origin by DIA mass spectrometry (n = 5). **Statistics:** Data are shown as **b-c)** mean ± SEM or **e-i)**: median (min to max) and p-values were calculated by **b), e-j)** unpaired two-tailed Student's t-test with or without Welch's correction **c)** one-way ANOVA for multiple comparisons with Šídák post-hoc test (Table S7). *$p \leq 0.05$, **$p \leq 0.01$, ****$p \leq 0.0001$, ns = not significant

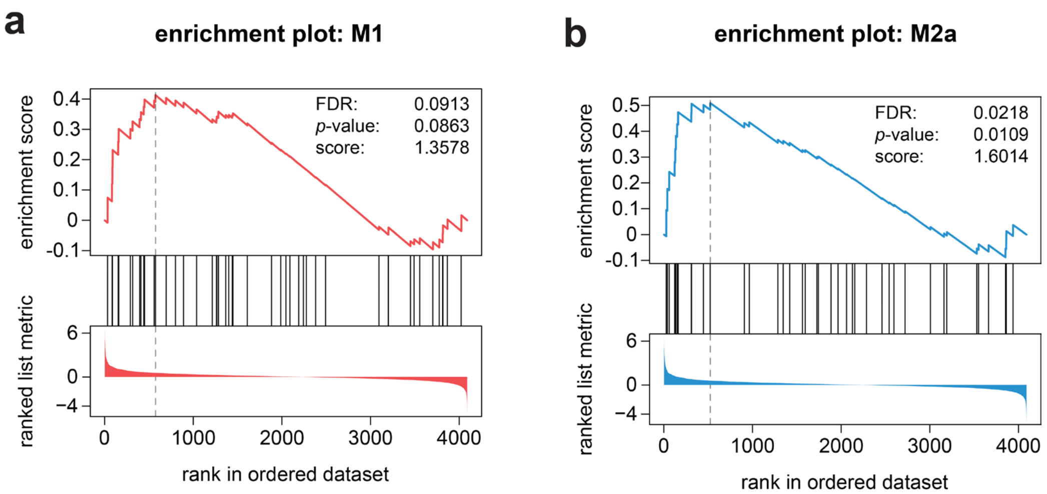

a
enrichment plot: M1
enrichment score
ranked list metric
FDR: 0.0913
p-value: 0.0863
score: 1.3578
rank in ordered dataset
b
enrichment plot: M2a
enrichment score
ranked list metric
FDR: 0.0218
p-value: 0.0109
score: 1.6014
rank in ordered dataset

Figure S2: Aging elicits neither a M1- nor M2-phenotypic switch in resident PM.

Gene set enrichment analysis (GSEA) was performed for the proteome of adult and old PM using the WEB-based GEne SeT AnaLysis Toolkit (WebGestalt). Enrichment plots are given for **a)** M1- and **b)** M2a-specific marker sets. The complete list of used markers is given in Table S2. Parameters for the enrichment analysis were as follows: minimum number of IDs in the category: 10, maximum number of IDs in the category: 5000, significance level: FDR < 0.1, number of permutation: 1000. **Statistics:** *p*-values, FDR and enrichment score were calculated by WebGestalt (Liao et al., 2019).

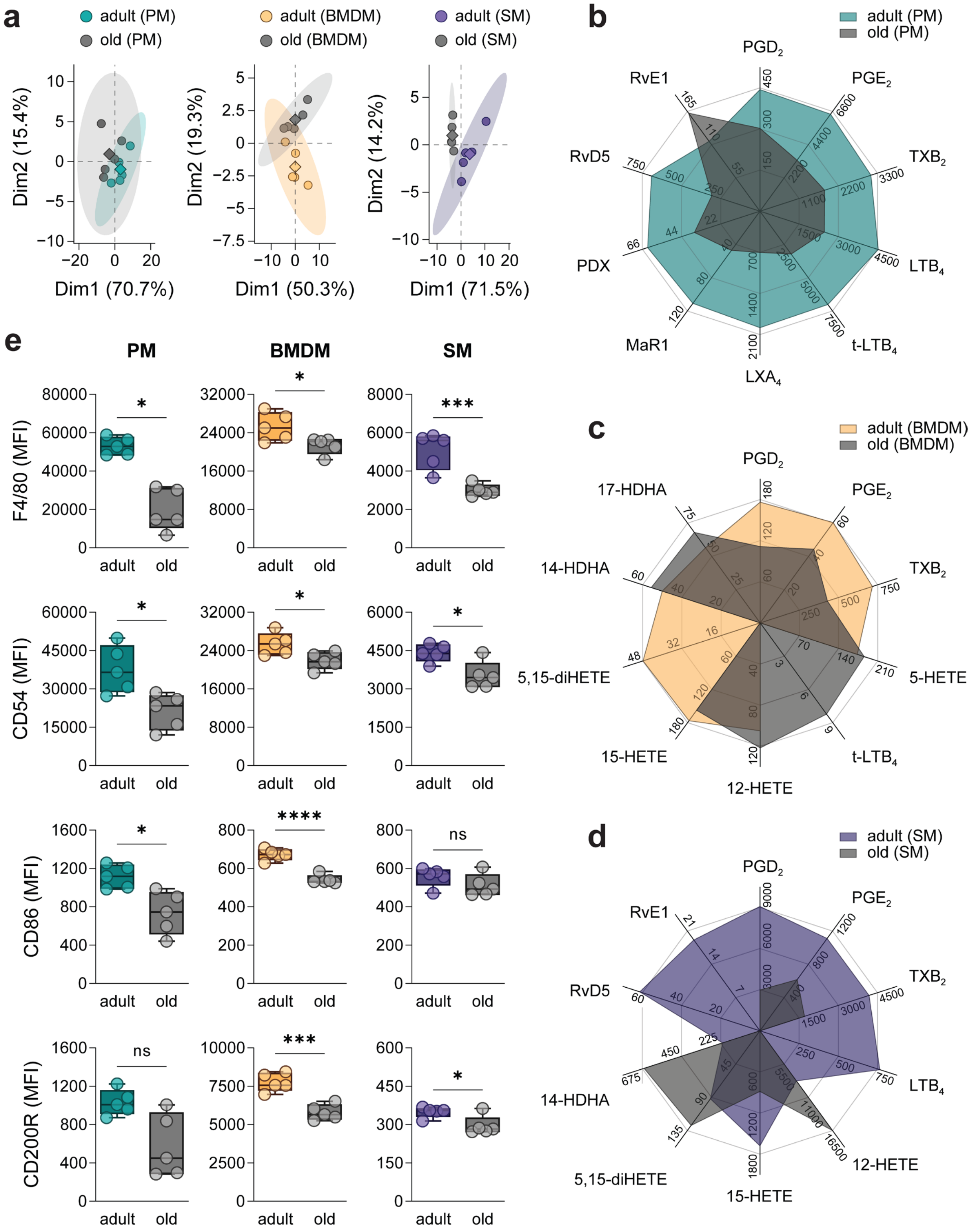

a
adult (PM)
old (PM)
adult (BMDM)
old (BMDM)
adult (SM)
old (SM)
Dim2 (15.4%)
Dim1 (70.7%)
Dim2 (19.3%)
Dim1 (50.3%)
Dim2 (14.2%)
Dim1 (71.5%)
b
adult (PM)
old (PM)
PGD2
PGE2
TXB2
LTB4
t-LTB4
LXA4
MaR1
PDX
RvD5
RvE1
c
adult (BMDM)
old (BMDM)
PGD2
PGE2
TXB2
5-HETE
t-LTB4
12-HETE
15-HETE
5,15-diHETE
14-HDHA
17-HDHA
d
adult (SM)
old (SM)
PGD2
PGE2
TXB2
LTB4
12-HETE
15-HETE
5,15-diHETE
14-HDHA
RvD5
RvE1
e
PM
BMDM
SM
F4/80 (MFI)
CD54 (MFI)
CD86 (MFI)
CD200R (MFI)
adult
old
*
***
****
ns

Figure S3: Age-related attenuation of macrophage responses persists across immunologic niches.

a) PCA of the metabololipidomic profiles of PM, BMDM and SM from adult and old mice after *E. coli* infection (Table S6; n = 5). **b)** Radar chart of selected highly abundant bioactive LM released by PM, BMDM and SM of adult and old origin (Table S6; n = 5; unit: pg/mL). **c)** Expression of F4/80, CD54, CD86 and CD200R in PM, BMDM and SM from adult and old mice was determined by flow cytometry and is given as MFI of all replicates (n = 5). **Statistics:** Data are shown as **b)** mean or **c)** median (min to max) and p-values were calculated by unpaired two-tailed Student's t-test with or without Welch's correction (Table S7). *$p \leq 0.05$, ***$p \leq 0.001$, ****$p \leq 0.0001$, ns = not significant

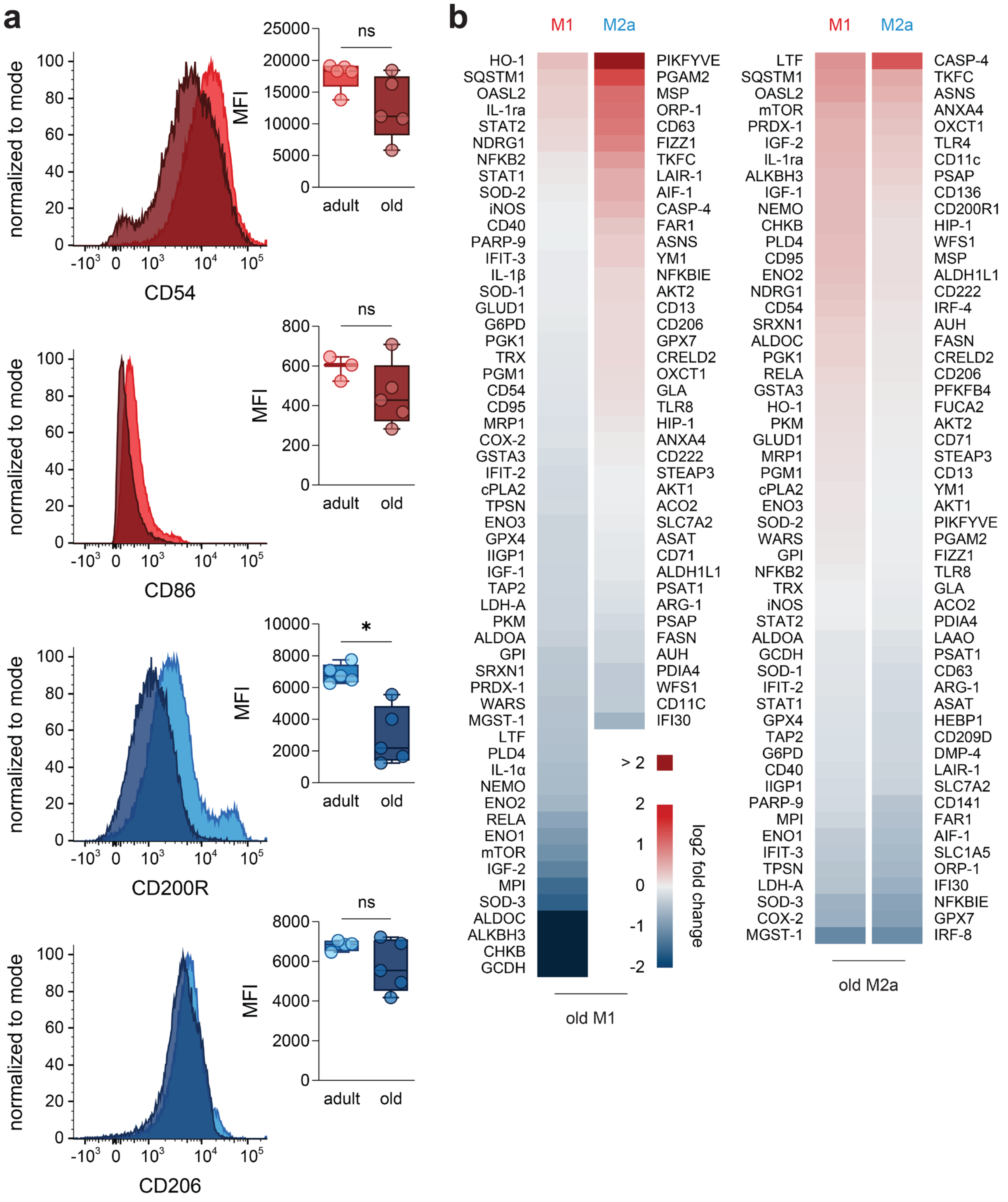
a
normalized to mode
CD54
CD86
CD200R
CD206
MFI
adult
old
ns
*
b
M1
M2a
old M1
old M2a
log2 fold change
> 2
HO-1
SQSTM1
OASL2
IL-1ra
STAT2
NDRG1
NFKB2
STAT1
SOD-2
iNOS
CD40
PARP-9
IFIT-3
IL-1β
SOD-1
GLUD1
G6PD
PGK1
TRX
PGM1
CD54
CD95
MRP1
COX-2
GSTA3
IFIT-2
cPLA2
TPSN
ENO3
GPX4
IIGP1
IGF-1
TAP2
LDH-A
PKM
ALDOA
GPI
SRXN1
PRDX-1
WARS
MGST-1
LTF
PLD4
IL-1α
NEMO
ENO2
RELA
ENO1
mTOR
IGF-2
MPI
SOD-3
ALDOC
ALKBH3
CHKB
GCDH
PIKFYVE
PGAM2
MSP
ORP-1
CD63
FIZZ1
TKFC
LAIR-1
AIF-1
CASP-4
FAR1
ASNS
YM1
NFKBIE
AKT2
CD13
CD206
GPX7
CRELD2
OXCT1
GLA
TLR8
HIP-1
ANXA4
CD222
STEAP3
AKT1
ACO2
SLC7A2
ASAT
CD71
ALDH1L1
PSAT1
ARG-1
PSAP
FASN
AUH
PDIA4
WFS1
CD11C
IFI30
LTF
SQSTM1
OASL2
mTOR
PRDX-1
IGF-2
IL-1ra
ALKBH3
IGF-1
NEMO
CHKB
PLD4
CD95
ENO2
NDRG1
CD54
SRXN1
ALDOC
PGK1
RELA
GSTA3
HO-1
PKM
GLUD1
MRP1
PGM1
cPLA2
ENO3
SOD-2
WARS
GPI
NFKB2
TRX
iNOS
STAT2
ALDOA
GCDH
SOD-1
IFIT-2
STAT1
GPX4
TAP2
G6PD
CD40
IIGP1
PARP-9
MPI
ENO1
IFIT-3
TPSN
LDH-A
SOD-3
COX-2
MGST-1
CASP-4
TKFC
ASNS
ANXA4
OXCT1
TLR4
CD11c
PSAP
CD136
CD200R1
HIP-1
WFS1
MSP
ALDH1L1
CD222
IRF-4
AUH
FASN
CRELD2
CD206
PFKFB4
FUCA2
AKT2
CD71
STEAP3
CD13
YM1
AKT1
PIKFYVE
PGAM2
FIZZ1
TLR8
GLA
ACO2
PDIA4
LAAO
PSAT1
CD63
ARG-1
ASAT
HEBP1
CD209D
DMP-4
LAIR-1
SLC7A2
CD141
FAR1
AIF-1
SLC1A5
ORP-1
IFI30
NFKBIE
GPX7
IRF-8

Figure S4: Aging compromises the polarization of macrophages on a proteomic level. Expression of **a)** CD54, CD86 on M1-PM and CD200R, CD206 on M2a-PM from adult and old origin was determined by flow cytometry (*n* = 3-5). Results are shown in representative histograms for each surface markers with mean fluorescence intensity (MFI) of all replicates. **b)** Heatmap showing age-related changes in the expression of M1 and M2a proteomic markers in M1- and M2a-PM (48 h) from old mice in comparison to adult; data acquired by DIA mass spectrometry (*n* = 5). Fold change is implicated by colour scale. **Statistics:** Data are shown as **a)** median (min to max) or **b)** median and *p*-values were calculated by unpaired two-tailed Student's *t*-test with or without Welch's correction (Table S7).

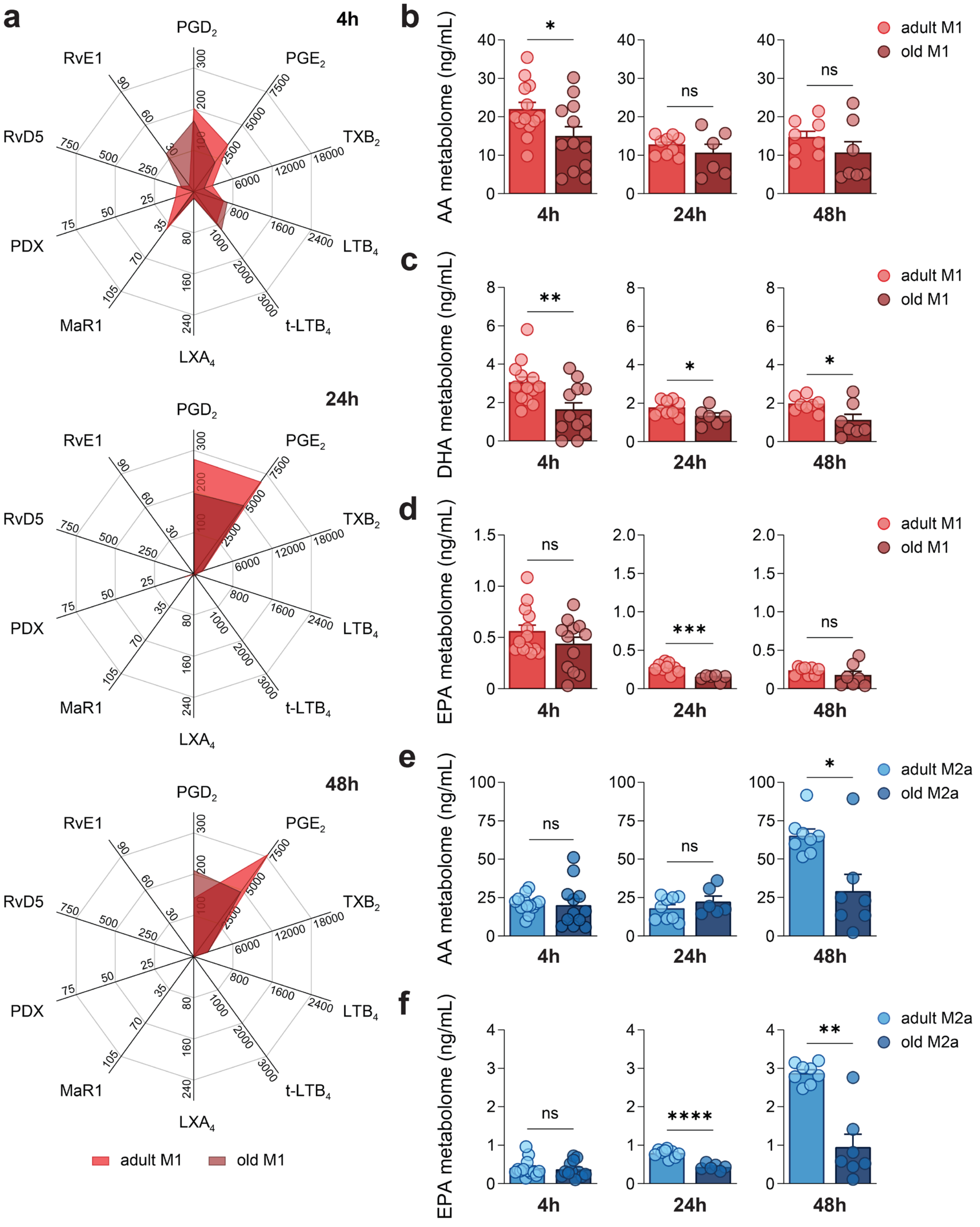

a
4h
24h
48h
PGD2
PGE2
TXB2
LTB4
t-LTB4
LXA4
MaR1
PDX
RvD5
RvE1
adult M1
old M1
b
AA metabolome (ng/mL)
c
DHA metabolome (ng/mL)
d
EPA metabolome (ng/mL)
e
AA metabolome (ng/mL)
adult M2a
old M2a
f
EPA metabolome (ng/mL)
ns

Figure S5: Aging has minor effects on the metabololipidome of M1 macrophages after *E. coli* infection.

Polarized PM (4 – 48 h) were infected with pathogenic *E. coli* for 90 min at a MOI of 50 and subsequent LM levels were determined by mass spectrometry (Table S6; 4 h: n = 12-13; 24 h: n = 6-9; 48 h: n = 7-8). **a)** Radar chart of selected bioactive LM released by M1-PM of adult and old mice (unit: pg/mL). Fatty acid metabolomes are given as sum of all metabolites from **b)** AA, **c)** DHA and **d)** EPA released by M1-PM of adult and old mice.

Statistics: Data are given as **b)** mean or **c)**, **d)** mean ± SEM and p-values were calculated by unpaired two-tailed Student's t-test with or without Welch's correction (Table S7). *$p \leq 0.05$, **$p \leq 0.01$, ***$p \leq 0.001$, ****$p \leq 0.0001$, ns = not significant

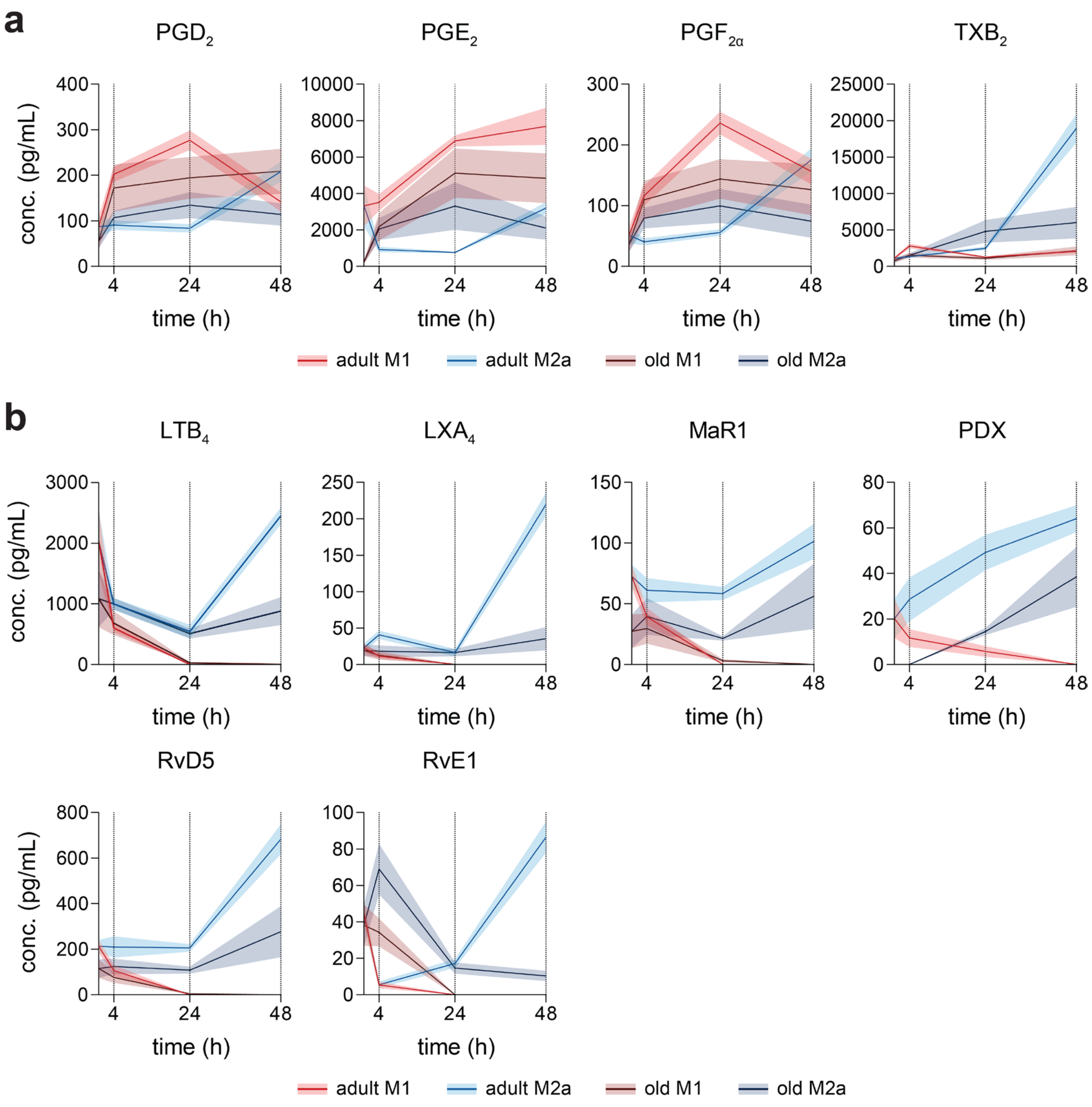
a
PGD2
PGE2
PGF2α
TXB2
conc. (pg/mL)
time (h)
adult M1
adult M2a
old M1
old M2a
b
LTB4
LXA4
MaR1
PDX
RvD5
RvE1
conc. (pg/mL)
time (h)
adult M1
adult M2a
old M1
old M2a

Figure S6: Aging causes a temporal shift in the release of bioactive LM after *E. coli* infection.

Polarized PM (4 – 48 h) were infected with pathogenic *E. coli* for 90 min at a MOI of 50 and subsequently LM levels were determined by UPLC-MS-MS (Table S6; 4 h: n = 12-13; 24 h: n = 6-9; 48 h: n = 7-8). Temporal release curves are given for **a**) COX- and **b**) LOX-derived bioactive eicosanoids and docosanoids from adult and old M1- and M2a-PM. **Statistics:** Data are shown as means ± SEM (indicated by error envelopes) and p-values (not shown) were calculated by unpaired two-tailed Student's t-test with or without Welch's correction (Table S7).

a

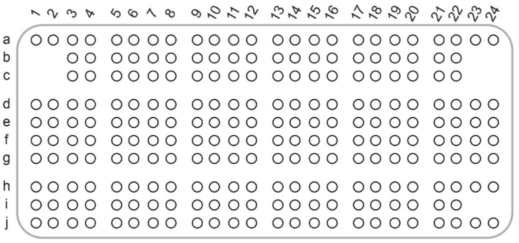

b

**spot**	**legend**	spot	legend
A1-A2	reference spots	F1-F2	IGFBP-3
A3-A4	adiponectin	F3-F4	IGFBP-5
A5-A6	amphiregulin	F5-F6	IGFBP-6
A7-A8	angiopoietin-1	F7-F8	IL-1α
A9-A10	angiopoietin-2	F9-F10	IL-1β
A11-A12	angiopoietin-like 3	F11-F12	IL-1ra
A13-A14	CD257	F13-F14	IL-2
A15-A16	CD93	F15-F16	IL-3
A17-A18	MCP-1	F17-F18	IL-4
A19-A20	MIP-1α/β	F19-F20	IL-5
A21-A22	RANTES	F21-F22	IL-6
A23-A24	reference spots	F23-F24	IL-7
B3-B4	MRP-1	G1-G2	IL-10
B5-B6	eotaxin	G3-G4	IL-11
B7-B8	MCP-5	G5-G6	IL-12
B9-B10	TARC	G7-G8	IL-13
B11-B12	MIP-3β	G9-G10	IL-15
B13-B14	MIP-3α	G11-G12	IL-17A
B15-B16	SCYA21	G13-G14	IL-22
B17-B18	MDC	G15-G16	IL-23
B19-B20	CD14	G17-G18	IL-27
B21-B22	CD40	G19-G20	IL-28A/B
C3-C4	CD160	G21-G22	IL-33
C5-C6	chemerin	G23-G24	LDL R
C7-C8	chitinase 3-like 1	H1-H2	leptin
C9-C10	CD142	H3-H4	LIF
C11-C12	C5a	H5-H6	lipocalin-2
C13-C14	complement factor D	H7-H8	LIX
C15-C16	CRP	H9-H10	M-CSF
C17-C18	fractalkine	H11-H12	MMP-2
C19-C20	KC	H13-H14	MMP-3
C21-C22	MIP-2	H15-H16	MMP-9
D1-D2	MIG	H17-H18	myeloperoxidase
D3-D4	IP-10	H19-H20	osteopontin
D5-D6	I-TAC	H21-H22	osteoprotegerin
D7-D8	BCA-1	H23-H24	gliostatin
D9-D10	CXCL16	I1-I2	PDGF-BB
D11-D12	cystatin C	I3-I4	pentraxin 2
D13-D14	DKK-1	I5-I6	pentraxin 3
D15-D16	CD26	I7-I8	periostin
D17-D18	EGF	I9-I10	Pref-1
D19-D20	endoglin	I11-I12	proliferin
D21-D22	endostatin	I13-I14	PCSK9
D23-D24	fetuin A	I15-I16	RAGE
E1-E2	FGF acidic	I17-I18	RBP4
E3-E4	FGF-21	I19-I20	Reg3G
E5-E6	Flt-3 ligand	I21-I22	resistin
E7-E8	Gas 6	J1-J2	reference spots
E9-E10	G-CSF	J3-J4	E-selectin
E11-E12	GDF-15	J5-J6	P-selectin
E13-E14	GM-CSF	J7-J8	serpin E1
E15-E16	HGF	J9-J10	serpin F1
E17-E18	ICAM-1	J11-J12	thrombopoietin
E19-E20	IFN-γ	J13-J14	TIM-1
E21-E22	IGFBP-1	J15-J16	TNF-α
E23-E24	IGFBP-2	J17-J18	VCAM-1
		J19-J20	VEGF
		J21-J22	WISP-1
		J23-J24	negative control

c

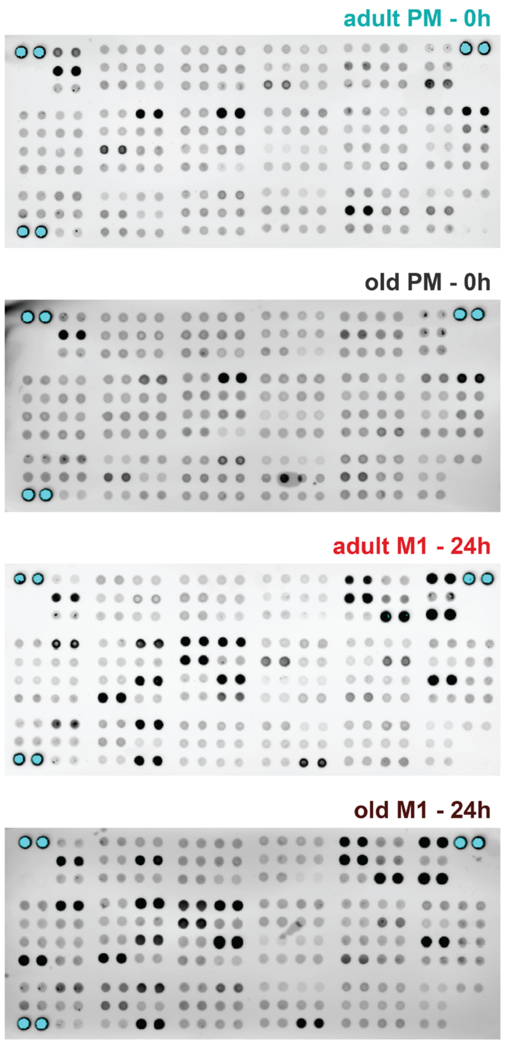

Figure S7: Raw data of cytokine screening of M1 macrophages during polarization.

a) Schematic layout of the cytokine array used for cytokine and chemokine screening of supernatants from adult and old M1-PM during the polarization (up to 48 h). **b)** Assignment of the spot on the cytokine array to their corresponding cytokines or chemokines. **c)** Representative pictures of cytokine arrays for pooled samples of adult and old PM at 0 h and 24 h of polarization towards M1. Raw values are given in Table S4.

Supplementary references

Liao, Y., Wang, J., Jaehnig, E. J., Shi, Z., & Zhang, B. (2019). WebGestalt 2019: gene set analysis toolkit with revamped UIs and APIs. *Nucleic Acids Res*, *47*(W1), W199-W205. https://doi.org/10.1093/nar/gkz401
